# Inter-laboratory comparison of eleven quantitative or digital PCR assays for detection of proviral bovine leukemia virus in blood samples

**DOI:** 10.1186/s12917-024-04228-z

**Published:** 2024-08-26

**Authors:** Aneta Pluta, Juan Pablo Jaworski, Casey Droscha, Sophie VanderWeele, Tasia M. Taxis, Stephen Valas, Dragan Brnić, Andreja Jungić, María José Ruano, Azucena Sánchez, Kenji Murakami, Kurumi Nakamura, Rodrigo Puentes, MLaureana De Brun, Vanesa Ruiz, Marla Eliana Ladera Gómez, Pamela Lendez, Guillermina Dolcini, Marcelo Fernandes Camargos, Antônio Fonseca, Subarna Barua, Chengming Wang, Aleksandra Giza, Jacek Kuźmak

**Affiliations:** 1 Department of Biochemistry, National Veterinary Research Institute, Puławy, 24-100 Poland; 2 Instituto de Virología E Innovaciones Tecnológicas (IVIT), Centro de Investigaciones en Ciencias Veterinarias y Agronómicas (CICVyA), Instituto Nacional de Tecnología Agropecuaria (INTA) - CONICET, Buenos Aires, Argentina; 3 CentralStar Cooperative, 4200 Forest Rd, Lansing, MI 48910 USA; 4 Department of Animal Science, College of Agriculture and Natural Resources, Michigan State University, East Lansing, Michigan, 48824 USA; 5 Niort Laboratory, Unit Pathology and Welfare of Ruminants, French Agency for Food, Environmental and Occupational Health and Safety (Anses), Ploufragan-Plouzané, Niort, France; 6 Croatian Veterinary Institute, Savska Cesta 143, Zagreb, 10000 Croatia; 7 Laboratorio Central de Veterinaria (LCV), Ministry of Agriculture, Fisheries and Food, Carretera M-106 (Km 1,4), Madrid, Algete 28110 Spain; 8 Department of Veterinary Sciences, Faculty of Agriculture, Iwate University, 3-18-8 Ueda, Morioka, 020-8550 Japan; 9 Departamento de Patobiología, Facultad de Veterinaria, Unidad de Microbiología, Universidad de La República, Ruta 8, Km 18, Montevideo, 13000 Uruguay; 10 Laboratorio de Virología, Departamento SAMP, Centro de Investigación Veterinaria de Tandil-CIVETAN (CONICET/UNCPBA/CICPBA), Buenos Aires, Argentina; 11 Laboratório Federal de Defesa Agropecuária de Minas Gerais, Pedro Leopoldo, Brazil; 12 Department of Pathobiology, College of Veterinary Medicine, Auburn University, Auburn, AL 36849-5519 USA; 13 Department of Omics Analyses, National Veterinary Research Institute, 24-100, Puławy, Poland

**Keywords:** Bovine leukemia virus** (**BLV), Quantitative real-time PCR (qPCR), Proviral DNA, DdPCR, BLV international network, Update on the efforts in harmonization qPCR

## Abstract

Bovine leukemia virus (BLV) is the etiological agent of enzootic bovine leukosis and causes a persistent infection that can leave cattle with no symptoms. Many countries have been able to successfully eradicate BLV through improved detection and management methods. However, with the increasing novel molecular detection methods there have been few efforts to standardize these results at global scale. This study aimed to determine the interlaboratory accuracy and agreement of 11 molecular tests in detecting BLV. Each qPCR/ddPCR method varied by target gene, primer design, DNA input and chemistries. DNA samples were extracted from blood of BLV-seropositive cattle and lyophilized to grant a better preservation during shipping to all participants around the globe. Twenty nine out of 44 samples were correctly identified by the 11 labs and all methods exhibited a diagnostic sensitivity between 74 and 100%. Agreement amongst different assays was linked to BLV copy numbers present in samples and the characteristics of each assay (i.e., BLV target sequence). Finally, the mean correlation value for all assays was within the range of strong correlation. This study highlights the importance of continuous need for standardization and harmonization amongst assays and the different participants. The results underscore the need of an international calibrator to estimate the efficiency (standard curve) of the different assays and improve quantitation accuracy. Additionally, this will inform future participants about the variability associated with emerging chemistries, methods, and technologies used to study BLV. Altogether, by improving tests performance worldwide it will positively aid in the eradication efforts.

**Supplementary Information:**

The online version contains supplementary material available at 10.1186/s12917-024-04228-z.

## Introduction

Bovine leukemia virus (BLV) is a deltaretrovirus from the *Orthoretrovirinae* subfamily of the *Retroviridae* family. An essential step in the BLV replication cycle is the integration of DNA copy of its RNA genome into the DNA of a host cell [1]. Once integrated, the proviral DNA is replicated along with the host’s DNA during cellular divisions, as for any cellular gene. The BLV is the etiologic agent of enzootic bovine leukosis (EBL). BLV causes a persistent infection in cattle, and in most cases this infection is asymptomatic [2]. In one-third of infected animals the infection progresses to a state of persistent lymphocytosis, and in 1 to 10% of infected cattle it develops into lymphosarcoma [2]. BLV induces high economic losses due to trade restrictions, replacement cost, reduced milk production, immunosuppression, and increased susceptibility to pneumonia, diarrhea, mastitis, and so on [3–6]. BLV is globally distributed with a high prevalence, except for Western Europe and Oceania, where the virus has been successfully eradicated through detection and elimination of BLV-infected animals [7, 8]. The agar gel immunodiffusion and ELISA for the detection of BLV-specific antibodies in sera and milk are the World Organization for Animal Health (WOAH, founded as OIE) prescribed tests for serological diagnosis but ELISA, due to its high sensitivity and ability to test many samples at a very low cost, is highly recommended [9]. Despite the advantages of serologic testing, there are some scenarios in which direct detection of the BLV genomic fragment was important to improve BLV detection. The most frequent cases is the screening of calves with maternal antibodies, acute infection, animals without persistent antibody response and animal subproducts (i.e., semen). In this regard, nucleic acid amplification tests such as real-time quantitative PCR (qPCR) allows for a rapid and highly sensitive detection of BLV proviral DNA (BLV DNA) that can be used to test infected and asymptomatic animals, before the elicitation of anti-BLV specific antibodies and when proviral load (PVL) are still low [10]. Furthermore, qPCR assays can serve as confirmatory tests for the clarification of inconclusive and discordant serological test results usually associated with these cases [11]. For these reasons, the inclusion of qPCR in combination with other screening tests might increase control programs efficiency. Additionally, qPCR allows the estimation of BLV PVL which is important for studying the dynamics of BLV infection (i.e., basic research). Further, considering that BLV PVL correlates with the risk of BLV transmission, this feature of qPCR can be exploited for developing rational segregation programs [12, 13]. The results of Kobayashi et al. suggest that high PVL is also a significant risk factor for progression to EBL and should therefore be used as a parameter to identify cattle for culling from the herd well before EBL progression [14]. Several qPCRs have been developed globally for the quantitation of BLV DNA. Although most assays have been properly validated by each developer, a proper standardization and harmonization of such tests is currently lacking. Considering that standardization and harmonization of qPCR methods and results are essential for comparisons of data from BLV laboratories around the world, this could directly impact international surveillance programs and collaborative research. We built a global collaborative network of BLV reference laboratories to evaluate the interlaboratory variability of different qPCRs and sponsored a harmonization of assays to hopefully impact international surveillance programs and research going forward.

In 2018 we conducted the first global trial of this kind to assess the interlaboratory variability of six qPCRs for the detection of BLV DNA [15]. Since this complex process is a continuous rather than a one-time effort, we now started a second study of this type. In this follow up study, we built a more comprehensive sample panel, accounting for a broader geographical diversification. Additionally, we increased the number of participants to ten collaborating laboratories plus one WOAH reference lab and tested novel methodologies including digital PCR (ddPCR) and FRET-qPCR. Finally, we established the next steps towards the international standardization of molecular assays for the detection of BLV DNA.

## Materials and methods

### Participants

The eleven laboratories that took part in the study were:(i) the Auburn University College of Veterinary Medicine (Auburn, Alabama, United States): (ii) AntelBio, a division of CentralStar Cooperative (Michigan, United States); (iii) Laboratórios Federais de Defesa Agropecuária de Minas Gerais (LFDA-MG, Pedro Leopoldo, Brasil); (iv) Centro de Investigación Veterinaria de Tandil (CIVETAN, Buenos Aires, Argentina); (v) the Faculty of Agriculture Iwate University (Iwate, Japan); (vi) Universidad de la República de Uruguay (UdelaR, Montevideo, Uruguay); (vii) the Croatian Veterinary Institute (Zagreb, Croatia); (viii) Instituto Nacional de Tecnología Agropecuaria (INTA, Buenos Aires, Argentina); (ix) Laboratorio Central de Veterinaria (LCV, Madrid, Spain); (x) the National Veterinary Research Institute (NVRI, Puławy, Poland) and (xi) the French Agency for Food, Environmental and Occupational Health and Safety (Anses, Niort, France). All European laboratories participating in this study are acting as national reference laboratories for EBL, NVRI acts as WOAH reference laboratory for EBL, while the remaining laboratories are nationally renowned entities for BLV diagnostics. The eleven participating methods are referred to below as qPCR1 – qPCR5, ddPCR6, qPCR7 – qPCR11, respectively.

### Sample collection and DNA extraction

A total of 42 DNA samples obtained from blood of naturally BLV-infected dairy cattle from Poland, Moldova, Pakistan, Ukraine, Canada and United States were used for this study. Thirty-six of them were archival DNA samples obtained between 2012–2018 as described in our previous studies on samples from Poland (*n* = 21) [16, 17], Moldova (*n* = 4) [18], Pakistan (*n* = 5) [19] and Ukraine (*n* = 6) [15, 20]. Between 2020–2021 6 peripheral blood and serum samples from naturally BLV-infected cattle were obtained from three dairy farms of Alberta, Canada and two dairy farms of Michigan, US. Serological testing and sample processing were conducted by the laboratories from which the samples originated. The genomic DNA from Canadian and US samples was extracted from whole blood using a Quick DNA Miniprep Plus kit (Zymo Research) and a DNeasy Blood & Tissue Kit (Qiagen), respectively in University of Calgary and Michigan State University and sent to the NVRI in the form of DNA solutions. Additionally, one plasmid DNA sample (pBLV344) was kindly supplied by Luc Willems (University of Liège, Belgium) and DNA extracted from FLK-BLV cells were included as positive controls. Finally, DNA extracted from PBL of a serologically negative cattle was included as negative control. At the NVRI, the DNA concentration in all samples was estimated by spectrophotometry using a NanoPhotometer (Implen). Each sample was divided into eleven identical aliquots containing between 800 and 4,000 ng of lyophilised genomic DNA. Eleven identical sets of these samples were lyophilized (Alpha 1–4 LSC basic, Martin Christ Gefriertrocknungsanlagen GmbH) and distributed to participating laboratories. At the NVRI, all samples were coded (identification [21] run numbers 1 to 44) to perform a blinded testing. The samples, together with instructions for their preparation (Additional file 1), were shipped by air at room temperature (RT).

### Examination of DNA quality/stability

Since different extraction methods and lyophilization process were employed for the preparation of the DNA samples, it was necessary to test the quality of the DNA at the NVRI laboratory. For that purpose, one complete set of samples (*n* = 44) was tested by Fragment Analyzer (Agilent Technologies), before and after freeze-drying, to assess DNA quality by calculating a Genomic Quality Number (GQN) for every sample. Low GQN value (< 2.5) represents sheared or degraded DNA. A high GQN (> 9) represents undegraded DNA. In addition, quality of DNA was assessed by determination of copy number of the histone H3 family 3A (*H3F3A*) housekeeping gene using quantitative real-time PCR (qPCR) [22]. The qPCR results were expressed as the number of *H3F3A* gene copies per 300 ng of DNA in each sample. Grubbs´ test was performed to determine outliers. To test the stability of DNA, samples were stored for 20 days at RT (10 days) and at + 4 °C (10 days) and were retested by Fragment Analyzer and qPCR 21 days later. A Mann–Whitney U-test was used to compare the median values between fresh and stored samples (time 0 and time 1), respectively.

### Description of BLV qPCR protocols used by participating laboratories

All participating laboratories performed their qPCR or ddPCR using a variety of different equipment, reagents, and reaction conditions, which had been set up, validated, and evaluated previously and are currently used as working protocols. The specific features of each of these protocols are described below and summarized in Table 1.

**Table 1:** Primers and probe for qPCR and ddPCR used in this study

PCR assay	Institution/ country	Detection method	Target	Primer/probe position	Sequence (5’-3’)	Amplicon size (bp)	Reference
qPCR1	AUCVM/US	FRET qPCR	*pol*	Fw, 2321–2345 Rv, 2484–2507 Anchor probe, 2407–2430 Reporter probe, 2431–2454	CCTCAATTCCCTTTAAACTAGAACG ATGGGCTTTGTAAGAGCATTTGTA GACGGGCCAGGCAATAATCCAGT-6-FAM LCRed640-TTCCCGGTACGGAAACCAAATGG-phosphate	187	[23, 24]
qPCR2	CentralStar/US	Multiplex TaqMan qPCR	*pol,* *β-actin,* spike-in control	N/A	N/A	N/A	[25]
qPCR3	LFDA-MG/ Brasil	TaqMan qPCR	LTR	BLV.LTR.114.Fw, 37–54 BLV.LTR.114.Rv, 131–150 BLV.LTR.114.Probe, 108–130	GCCCCGTAAACCAGACAG CTCAGGGTGTGGATTTCTCG FAM-TACCTCCCC/ZEN/AACTTCCCCTTTCC-IABkF	114	[26]
qPCR4	CIVETAN/Argentina	SYBRGreen qPCR	*pol*	Fw, 4618–4636 Rv, 4657–4676	CACCATTCACCCCCACTTG TCAGAGCCCTTGGGTGTTTC	59	[27]
qPCR5	FA-IU/Japan	Duplex TaqMan qPCR	*pol,* *RPPH1*	N/A	N/A	N/A	[28]
ddPCR6	UdelaR/Urugway	TaqMan ddPCR	*env*	Fw, 5384–5406 Rw, 5525–5546 Probe, 5477–5497	CAGTGACTGGGTTCCCTCTGTC AGGGCGAGRCCGGGTCCAGAG HEX-CCCTCCCTGGGCTCCCGARA-BHQ1	162	[29]
qPCR7	VEINST/Croatia	TaqMan qPCR	*pol*	MRBLVL Fw, 2321–2340 MRBLVR Rv, 2421–2440 MRBLV Probe, 2341–2360	CCTCAATTCCCTTTAAACTA GTACCGGGAAGACTGGATTA 6FAM-GAACGCCTCCAGGCCCTTCA-BHQ1	120	[30]
qPCR8	INTA/Argentina	SYBRGreen qPCR	*pol*	BLVpol5f Fw, 2321–2340 BLVpol3r Rv, 2421–2440	CCTCAATTCCCTTTAAACTA GTACCGGGAAGACTGGATTA	120	[30, 31]
qPCR9	LCV/Spain	Duplex TaqMan qPCR	*pol* *β-actin*	MRBLVL Fw, 2321–2340 MRBLVR Rv, 2421–2440 Probe, 2341–2360 ACT-1005-F Fw, 1005–1029 ACT-1135-R Rv, 1135–1114 ACT-1081 Probe, 1081–1105	CCTCAATTCCCTTTAAACTA GTACCGGGAAGACTGGATTA Cy5-GAACGCCTCCAGGCCCTTCA-BHQ1 CAGCACAATGAAGATCAAGATCATC CGGACTCATCGTACTCCTGCTT HEX-TCGCTGTCCACCTTCCAGCAGATGT-BHQ1	120 130	[30, 32]
qPCR10	NVRI/Poland	TaqMan qPCR	*pol*	MRBLVL Fw, 2321–2340 MRBLVR Rv, 2421–2440 MRBLV Probe, 2341–2360	CCTCAATTCCCTTTAAACTA GTACCGGGAAGACTGGATTA 6FAM-GAACGCCTCCAGGCCCTTCA-BHQ1	120	[30]
qPCR11	Anses/France	TaqMan qPCR	*pol*	MRBLVL Fw, 2321–2340 MRBLVR Rv, 2421–2440 MRBLV Probe, 2341–2360	CCTCAATTCCCTTTAAACTA GTACCGGGAAGACTGGATTA 6FAM-GAACGCCTCCAGGCCCTTCA-BHQ1	120	[30]

*N/A* no data available

All laboratories applied standard procedures for avoiding false-positive results indicative of DNA contamination, such as the use of separate rooms for preparing reaction mixtures, adding the samples, and performing the amplification reaction. One of the ten BLV qPCRs used LTR region and the remaining nine qPCRs used the *pol* gene as the target sequence for amplification, while the ddPCR amplified the *env* gene.

#### Method qPCR1

The BLV qPCR amplifying a 187-bp *pol* gene was performed according to a previously published methods [23, 24]. A real-time fluorescence resonance energy transfer (FRET) PCR was carried out in a 20-μl PCR mixture containing 10 μl handmade reaction master mix and 10 μl genomic DNA. The PCR buffer was 4.5 mM MgCl2, 50 mM KCl, 20 mM Tris–HCl, pH 8.4, supplemented with 0.05% each Tween20 and Non-idet P-40, and 0.03% acetylated BSA (Roche Applied Science). For each 20 μl total reaction volume, the nucleotides were used at 0.2 mM each and 1.5 U Platinum Taq DNA polymerase (Invitrogen, Carlsbad, CA, USA) was used. Primers were used at 1 μM, LCRed640 probe was used at 0.2 μM, and 6-FAM probe was used at 0.1 μM. Amplification was performed in the Roche Light Cycler 480 II (Roche Molecular Biochemicals) using 10 min denaturation step at 95 °C, followed by 18 high-stringency step-down thermal cycles and 30 low-stringency fluorescence acquisition cycles.

A plasmid containing the BLV-PCR amplicon region was diluted ten-fold from 1 × 10^5^ copies to 10 copies per 10 µl and was used as a standard to measure the BLV copy numbers.

#### Method qPCR2

A BLV proviral load qPCR assay developed by AntelBio, a division of CentralStar Cooperative Inc. on Applied Biosystems 7500 Real-Time PCR system [25, 33]. This multiplex assay amplifies the BLV *pol* gene along with the bovine *β-actin* gene and an internal amplification control, “Spike”. A quantitative TaqMan PCR was carried out in a 25-μl PCR mixture containing 12.5 µl of 2X InhibiTaq Multiplex HotStart qPCR MasterMix (Empirical Bioscience), 16 nM each BLV primer, 16 nM each *β-actin* primer, 8 nM each spike primer, 8 nM BLV FAM-probe, 8 nM *β-actin* Cy5-probe, 4 nM spike JOE-probe, 1 µl of an internal spike-in control (10,000 copies per µl), 7.25 µl of nuclease-free water and 4 µl of DNA sample for each qPCR reaction. The thermal PCR protocol was as follows: 95 °C for 10 min, 40 × (95 °C for 15 s, 60 °C for 1 min). Copy numbers of both the BLV *pol* gene and bovine *β-Actin* were derived using a plasmid containing target sequences, quantified by ddPCR, diluted 1 × 10^6^ copies per µl to 10 copies per µl in tenfold dilutions. DNA concentrations of each sample were measured using a Qubit 4 Fluorometer and used in combination with the qPCR copy numbers to calculate BLV copies per 100 ng.

#### Method qPCR3

The qPCR assays for the BLV LTR gene were performed according to a previously published methods [26]. Genomic DNA was amplified by TaqMan PCR with 10 μl of GoTaq Probe qPCR Master Mix × 2 (Promega), 0.6 pmol/μl each primer, 0.3 pmol/µl double-quenched probe and 100 ng genomic DNA. Amplification was performed in the CFX96 cycler (BioRad) according to the protocol: 5 min denaturation at 95°C followed by 45 cycles (60 s at 94°C and 60 s at 60°C). The efficiency of each reaction was calculated from the serial dilution of DNA extracted from BLV persistently infected fetal lamb kidney (FLK) cells, starting at a concentration of 100 ng/µl [21]. The detection limit was tested using a plasmid containing the target of the qPCRs, starting at 10^3^ ng/µl.

#### Method qPCR4

The quantitative real-time PCR was done with the primers for the BLV *pol* gene as previously described [34]. The qPCR reaction mix contained 1 × PCR Master Mix with SYBR Green (FastStart Universal SYBR Green Master Rox, Roche), 0.3 μM each primer and 30 ng of extracted genomic DNA. Amplification was performed in QuantStudio 5 Real-Time PCR System (Applied Biosystems) under the following conditions: 2 min at 50 °C, 10 min at 95 °C, 40 cycles of 15 s at 95 °C and 60 s at 60 °C. A standard curve of six tenfold serial dilutions of pBLV, containing 1 × 10^6^ to 10 BLV copies, was built and run 3 times for validation of the method. The number of provirus copies per reaction (100 ng) was calculated.

#### Method qPCR5

BLV PVLs were determined by using qPCR kit, RC202 (Takara Bio, Shiga, Japan) [28, 35]. This qPCR assay amplifies the BLV *pol* gene along with the bovine *RPPH1* gene as an internal control. Briefly, 100 ng genomic DNA was amplified by TaqMan PCR with four primers for *pol* gene and *RPPH1* gene according to the manufacturer’s instructions: 30 s denaturation at 95 °C followed by 45 cycles (5 s at 95 °C and 30 s at 60 °C). The qPCR was performed on a QuantStudio 3 Real-Time PCR System (Thermo Fisher Scientific K.K., Tokyo, Japan). Standard curve was generated by creating tenfold serial dilutions of the standard plasmid included in the kit. The standards for calibration ranged from 1 to 10^6^ copies/reaction and were run in duplicate. The number of provirus copies per 100 ng was calculated.

#### Method ddPCR6

The digital droplet PCR (ddPCR) assay for the *env* gene of the BLV was performed using the protocol previously described by [28, 29]. An absolute quantification by TaqMan ddPCR was performed in a typical 20-μl assay, 1 μl of DNA sample was mixed with 1 μl of each primer (10 μM), 0.5 μl of probe (10 μM), and 2 × Supermix emulsified with oil (Bio-Rad). The droplets were transferred to a 96-well plate (Eppendorf). The PCR assay was performed in a thermocycler (C1000 touch cycler; Bio-Rad) with the following parameters: initial denaturation of 10 min at 95 °C, then 40 cycles of 30 s at 94 °C, and 1 min at 58 °C, with final deactivation of the enzyme for 10 min at 98 °C. The presence of fluorescent droplets determined the number of resulting positive events that were analyzed in the software (QuantaSoft v.1.7.4; Bio-Rad), using dot charts. The number of provirus copies per 100 ng were calculated. Each sample was run in duplicate, and results were averaged.

#### Method qPCR7

This qPCR method for the BLV *pol* gene is a modified option of widely available quantitative TaqMan qPCR described by Rola-Łuszczak et al. [11], using the same primers and standards. A quantitative TaqMan PCR was performed in a 20 μl PCR mix containing 10 μl of 2 × ORA qPCR Probe ROX L Mix (highQu, Kraichtal, Germany), 2 μl primer/probe mix (final concentration 400 nM of each of the primers, 200 nM of BLV probe), and 3 μl extracted genomic DNA. Amplification was performed in the Rotor-Gene Q system (Qiagen) with an initial denaturation step and polymerase activation at 95 °C for 3 min, followed by 45 cycles of 95 °C for 5 s and 60 °C for 30 s. As a standard, plasmid pBLV1 (NVRI, Pulawy, PL) containing a BLV *pol* fragment was used. Tenfold dilutions of plasmid DNA were made from 1 × 10^10^ copies to 1 × 10^1^ copies per reaction and used to generate the standard curve and estimate BLV copy number per 100 ng.

#### Method qPCR8

Proviral load quantification was assessed by SYBR Green real-time quantitative PCR (qPCR) using the *pol* gene as the target sequence [36]. Briefly, 12-μl PCR mixture contained Fast Start Universal SYBR Green Master Mix (Roche), 800 nM each BLV *pol* primers and 1 µl DNA as template. The reactions were incubated at 50 °C for 2 min and 95 °C for 10 min, followed by 40 cycles at 95 °C for 15 s, 55 °C for 15 s and 60 °C for 1 min. All samples were tested in duplicate on a StepOne Plus machine (Applied Biosystems). A positive and negative control, as well as a no-template control, were included in each plate. After the reaction was completed, the specificity of the amplicons was checked by analyzing the individual dissociation curves. As a standard, plasmid pBLV1 (NVRI, Pulawy, PL) containing a BLV *pol* fragment was used. Tenfold dilutions of plasmid DNA were made from 1 × 10^6^ to 10 copies per µl and used to generate the standard curve and estimate BLV copy number per 100 ng.

#### Method qPCR9

This qPCR method is a modified option of widely available quantitative TaqMan qPCR described by Rola-Łuszczak et al. [11], using the same primers and standards. The detection of BLV genome was combined with an endogenous control system (Toussaint 2007) in a duplex assay. Briefly, 20-µl qPCR reaction contained AhPath ID™ One-Step RT-PCR Reagents with ROX (Applied Biosystems, CA, USA) – 10 µl of 2 × RT-PCR buffer and 0.8 µl of 25 × RT-PCR enzyme mix, 400 nM each primer for *pol* gene, 100 nM BLV specific probe, 40 nM each β-actin primer, 40 nM β-actin specific probe and 2 µl DNA sample. All samples were tested in ABI7500 Real-Time PCR System (Applied Biosystems) according to the following protocol: 10 min at 48 °C (reverse transcription), 10 min at 95 °C (inactivation reverse transcriptase / activation Taq polymerase) followed by 45 cycles (15 s at 95 °C and 60 s at 60 °C). As a standard, plasmid pBLV1 (NVRI, Pulawy, PL) containing a BLV *pol* fragment was used. Tenfold dilutions of plasmid DNA were made from 1 × 10^4^ copies to 0.1 copies per μl and used to generate the standard curve and estimate BLV copy number per 100 ng.

#### Method qPCR10

The BLV qPCR was performed as published previously [11]. A quantitative TaqMan PCR was carried out in a 25-μl PCR mixture containing 12.5 μl of 2 × QuantiTect Multiplex PCR NoROX master mix (Qiagen), 0.4 μM each primer, 0.2 μM specific BLV probe, and 500 ng of extracted genomic DNA. Amplification was performed in the Rotor-Gene Q system (Qiagen) using an initial denaturation step and polymerase activation at 95 °C for 15 min, followed by 50 cycles of 94 °C for 60 s and 60 °C for 60 s. All samples were amplified in duplicate. As a standard, the pBLV1 plasmid (NVRI, Pulawy, PL), containing a 120-bp BLV *pol* fragment, was used. Tenfold dilutions of this standard were made from 1 × 10^6^ copies per μl to 100 copies per μl and were used to estimate the BLV copy numbers per 100 ng.

#### Method qPCR11

This qPCR method for the BLV *pol* gene is a modified option of widely available quantitative TaqMan qPCR described by Rola-Łuszczak et al. [11], using the same primers and standards. The reaction mixture contained 400 nM of each primer, 200 nM of probe, 10 µl of 2 × SsoFast probes supermix (Bio-Rad), 5 µl of DNA sample and H_2_O up to 20 µl of the final volume. PCR assays were carried out on a CFX96 thermocycler (Bio-Rad) under the following amplification profile: 98 °C for 3 min, followed by 45 cycles of 95 °C for 5 s and 60 °C for 30 s. As a standard, plasmid pBLV1 (NVRI, Pulawy, PL) containing a BLV *pol* fragment was used. Tenfold dilutions of plasmid DNA were used to generate the standard curve and estimate BLV copy number per 100 ng.

### Analysis of BLV pol, env and LTR sequences targeted by particular qPCR/ddPCR assays

In order to assess full-length *pol*, *env* and LTR sequence variability among BLV genotypes, all BLV sequences (*n* = 2191) available on 30 September 2023 in GenBank (https://www.ncbi.nlm.nih.gov/GenBank/) repository were retrieved. From the collected sequences, 100 *pol*, *env* and LTR sequences, which were characterized by the highest level of sequence variability and divergence, were selected for the further analysis. A *pol*-based, *env*-based and LTR-based maximum likelihood (ML) phylogenetic trees (see Additional file 6) was constructed to assign genotypes to the unassigned BLV genomes [37–39]. For all genes and LTR region the Tamura-Nei model and Bootstrap replications (1,000) were applied. In this analysis, *pol* sequences were assigned to 7 BLV genotypes (G1, G2, G3, G4, G6, G9, and G10), while *env* and LTR sequences were assigned to 10 BLV genotypes (G1, G2, G3, G4, G5, G6, G7, G8, G9, and G10). Phylogeny of the same isolates assigned to particular genotypes by ML method was confirmed by Mr. Bayes analysis [40–42] (data not shown). From this analysis, a total of 100 full-length *pol, env* and LTR sequences were used for multiple-sequence alignment (MSA) using ClustalW algorithm, implemented in MEGA X. For all sequences, nucleotide diversity (π), defined as the average number of nucleotide differences per site between two DNA sequences in all possible pairs in the sample population, was estimated using MEGA X. To measure the relative variation in different positions of aligned genes and LTR region the Shannon’s entropy (a quantitative measure of diversity in the alignment, where H = 0 indicates complete conservation) was estimated using BioEdit v. 7.2.5 software 64. The statistical analyses were performed using DATAtab e.U. Graz, Austria and GraphPad Software by Dotmatics, Boston.

## Results

### Examination of the quality and stability of DNA samples

**Fig. 1:**
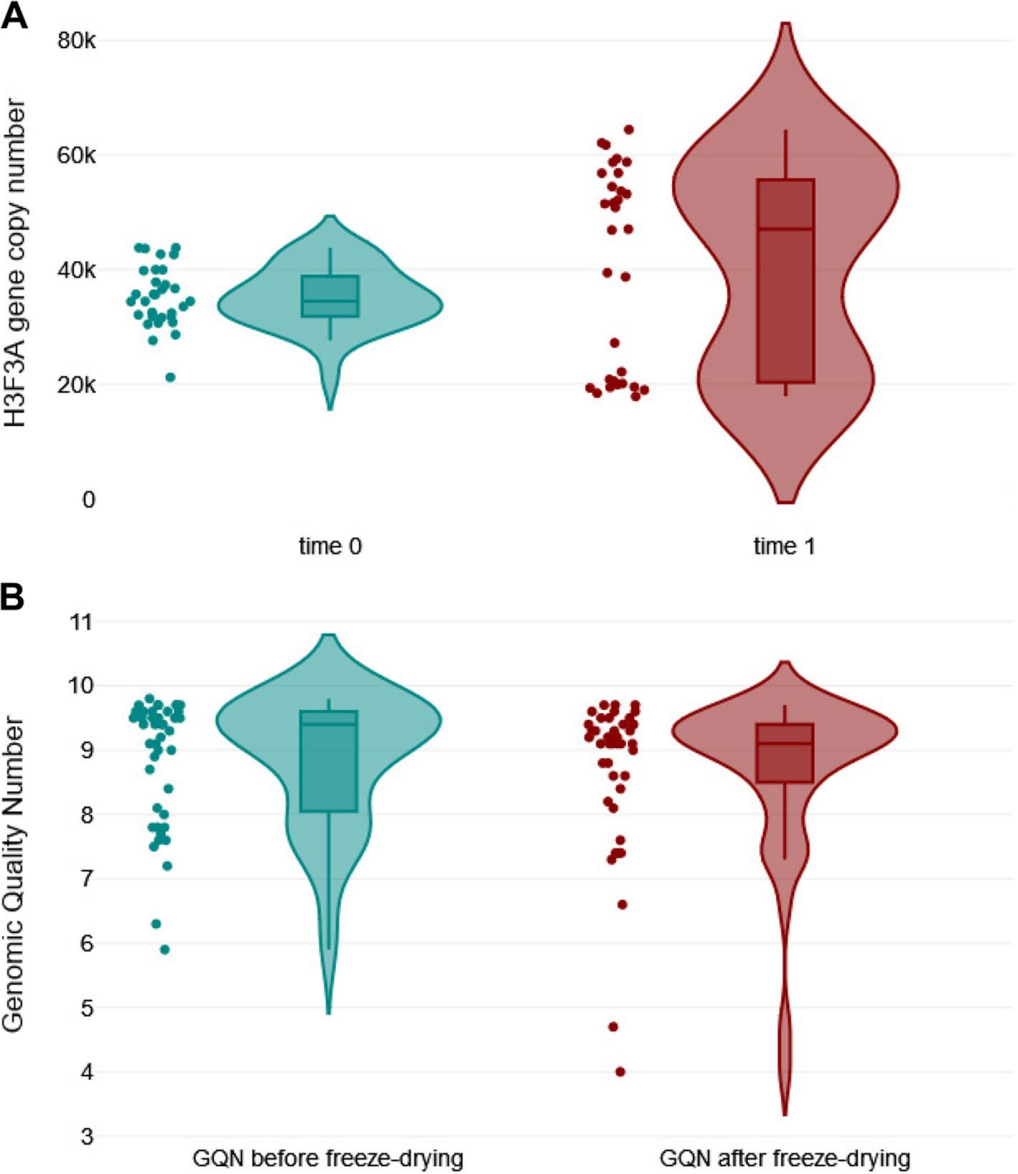
Assessment of the stability of DNA samples. **A** Shown are copy numbers of the H3F3A housekeeping gene in 43 DNA samples that were stored in 10 days at RT and 10 days at + 4°C and tested twice with a 21-day interval. A Mann–Whitney U-test was used to compare the median values between two groups (*P* = 0.187); **B** Shown are GQN values (*n* = 43) tested twice with a 21-day interval: `before freeze-drying` and `after freeze-drying`. A Mann–Whitney U-test results between two groups (*P* = 0.236)

To test the quality of DNA samples, the *H3F3A* copy number of each individual sample was assessed by qPCR at the NVRI. Copy numbers were normalized to DNA mass input and results were expressed as copy numbers per 300 ng of total DNA. The respective values were tested by Grubbs' test. The results for 43 DNA samples (sample ID: 42 with BLV genome plasmid was excluded) followed a normal distribution (Shapiro–Wilk 0.97; *P* = 0.286), with a mean value of 35,626 copies (95% confidence interval [43] 33,843 to 37,408 copies), a minimum value of 19,848 copies and a maximum value of 46,951 copies (see Additional file 2). Despite a low value for sample ID: 40 no significant outlier was detected in the dataset (*P* > 0.05). Therefore, it can be assumed that the DNA quality was acceptable for all samples present in the panel. Next, DNA stability was assessed by retesting the *H3F3A* copy numbers in each sample (*n* = 43) after a combined storage consisting in 10 days at RT and 10 days at + 4°C. A Mann–Whitney U-test was used to compare the median values between fresh and stored samples (time 0 and time 1, respectively), and no significant difference was observed at the 5% level (*P* = 0.187) (Fig. 1A).

In addition, the quality of DNA samples after lyophilization was analyzed. DNA from individual samples (*n* = 43) was assessed with the genomic DNA quality number on the Fragment Analyzer system. The GQN from all lyophilized samples ranged from 4.0 to 9.7—that represented undegraded DNA. There was no significant difference in GQN values between `before freeze-drying` and `after freeze-drying` groups with respect to the corresponding DNA samples (*P* = 0.236) (Fig. 1B). Altogether, these results suggested that sample storage, lyophilization and shipping has a minimal impact in DNA stability and further testing during the interlaboratory trial.

### Detection of BLV proviral DNA by different qPCR assays

A total of 44 DNA samples, including two positive (ID: 42 and 43) and one negative (ID: 32) controls, were blinded and independently tested by eleven laboratories using their own qPCR methods (Table 2). All laboratories measured the concentration of DNA in samples (Additional file 3). BLV provirus copy number was normalized to DNA concentration and expressed per 100 ng of genomic DNA for each test.

**Table 2:** Origin of the samples included in the study (*n*=44) and the respective results. Copy numbers were then normalized to DNA concentration and expressed as per 100 ng of genomic DNA

**Sample No.**	**Origin**	**Genotype**	**ELISA**	**qPCR result (no. of BLV DNA copies per 100 ng of total DNA)**^**a**^										
				**qPCR1** **(pol)**	**qPCR2** **(pol)**	**qPCR3** **(LTR)**	**qPCR4** **(pol)**	**qPCR5** **(pol)**	**ddPCR6** **(env)**	**qPCR7**^d^ **(pol)**	**qPCR8**^d^ **(pol)**	**qPCR9**^d^ **(pol)**	**qPCR10**^d^ **(pol)**	**qPCR11**^d^ **(pol)**
**1**	Poland	G4	+	17253.9	7820.2	962541.0	285583.3	3490.2	4480.0	36700.0	10820.0	24700.0	11613.8	84463.7
**2**	Poland	G4	+	2042.6	465.6	48555.9	19950.0	947.4	442.0	159.0	489.0	209.0	835.8	20127.2
**3**	Ukraine	G4	+	30365.9	8329.2	184258.8	398733.3	19476.0	8320.0	50800.0	28699.0	16400.0	13441.1	165171.6
**4**	Ukraine	G8	+	6994.0	4818.0	14457.6	120130.0	7180.4	4.4	5710.0	5459.0	3900.0	3384.3	52440.6
**5**	Ukraine	G8	+	6653.5	6354.8	64721.9	109896.7	4753.6	1734.0	7110.0	9199.0	1660.0	46.3	249756.1
**6**	Ukraine	^**b**^Ns	+	11586.5	10479.8	191854.7	217700.0	11272.9	1501.2	4970.0	12250.0	15500.0	9476.4	48900.5
**7**	Ukraine	Ns	+	89.1	52.3	43.3	5613.3	7.7	10.4	12.6	51.0	1.7	27.0	502.7
**8**	Poland	G8	+	1146.9	381.9	119.5	8993.3	479.7	NEG	173.0	267.0	118.0	388.9	2738.9
**9**	Poland	G7	+	0.3	NEG	NEG	NEG	1.0	NEG	12.3	17.0	NEG	NEG	6.1
**10**	Moldova	G7	+	130.5	1345.1	2533.8	7090.0	21.1	76.0	188.0	224.0	3.4	207.3	2935.0
**11**	Ukraine	Ns	+	1618.1	1274.6	308.8	18996.7	934.5	NEG	1480.0	948.0	209.0	816.1	8483.0
**12**	Moldova	G7	+	5964.5	6473.7	127613.4	235810.0	1673.2	5320.0	21600.0	11541.0	5950.0	16235.6	171017.9
**13**	Poland	G4	+	41743.1	2816.6	580303.9	378866.7	15555.0	6100.0	21300.0	16300.0	18400.0	28410.3	505675.6
**14**	Poland	Ns	+	2.0	NEG	NEG	NEG	2.5	NEG	11.0	4.0	0.1	2.1	97.9
**15**	Poland	G8	+	13.0	NEG	NEG	NEG	6.4	NEG	NEG	12.0	0.1	3.9	170.7
**16**	Moldova	Ns	+	6.8	NEG	14.1	NEG	4.0	4.6	40.8	6.0	0.1	1.8	97.8
**17**	Poland	G4	+	40.2	69.6	1002.7	736.7	11.3	12.0	18.8	18.0	1.2	19.8	368.3
**18**	Poland	G7	+	1.5	NEG	306.5	536.7	NEG	3.8	23.7	NEG	0.1	2.5	35.0
**19**	Poland	G7	+	18092.8	807.5	246292.1	432900.0	3215.4	7760.0	47700.0	16849.0	19800.0	18718.6	218610.3
**20**	Moldova	G7	+	2188.4	19195.8	268180.6	360833.3	2957.0	5700.0	29100.0	3863.0	985.0	19494.0	129483.2
**21**	Poland	G4	+	423.9	1593.3	295.7	8503.3	202.7	214.0	460.0	267.0	7.4	277.0	5211.4
**22**	Poland	G4	+	33005.6	14517.9	335322.0	364700.0	13565.7	12040.0	15400.0	4539.0	12200.0	28424.2	152574.9
**23**	Poland	G4	+	4776.8	4750.2	197.2	80110.0	3385.1	1800.0	13200.0	2050.0	1280.0	2269.0	71669.1
**24**	Poland	G7	+	4.9	NEG	81.6	NEG	1.9	8.2	34.1	5.0	0.1	1.4	99.2
**25**	Poland	G8	+	38.1	1152.2	8.0	533.3	30.0	NEG	144.0	26.0	0.2	33.8	524.7
**26**	Poland	G8	+	1017.4	921.7	233.9	19566.7	828.3	NEG	1850.0	850.0	74.4	781.9	9819.5
**27**	Poland	G4	+	41388.9	9513.7	NEG	218583.3	10410.8	4000.0	30800.0	6033.0	10900.0	18878.0	131461.4
**28**	Poland	G7	+	1.0	NEG	480.1	NEG	NEG	NEG	17.5	9.0	0.3	0.7	NEG
**29**	Poland	G4	+	23913.0	1886.5	173311.8	334533.3	16739.7	7570.0	12700.0	2476.0	7590.0	27163.2	170384.4
**30**	Poland	G8	+	7.8	113.5	NEG	326.7	4.8	NEG	6.3	12.0	1.0	3.8	41.5
**31**	Pakistan	G6	+	8920.5	3293.8	13751.5	57813.3	1633.2	860.0	3570.0	2386.0	825.0	3197.8	49053.1
**32**	Poland	^c^Na	-	^a^NEG	NEG	NEG	NEG	NEG	NEG	NEG	NEG	NEG	NEG	NEG
**33**	Pakistan	G6	+	1308.8	417.5	29221.6	15846.7	387.0	351.8	1490.0	800.0	103.0	856.7	18479.7
**34**	Pakistan	G1	+	1.6	NEG	259.8	NEG	NEG	NEG	NEG	8.0	0.1	0.7	NEG
**35**	Pakistan	G1	+	7000.0	1082.7	104381.2	76063.3	5485.9	1700.0	9480.0	4156.0	1200.0	3869.7	23367.2
**36**	Pakistan	G6	+	326.3	52.8	2742.6	5933.3	240.3	176.0	187.0	209.0	16.2	188.2	1499.4
**37**	Canada	G1	+	385.7	3083.5	437.2	9630.0	245.0	132.0	430.0	326.0	26.5	169.9	4185.5
**38**	Canada	G1	+	1452.8	84.4	78032.9	24483.3	911.6	338.0	2000.0	949.0	133.0	591.4	16415.2
**39**	Canada	G1	+	1245.8	871.8	6963.9	19863.3	667.6	268.0	2140.0	989.0	111.0	410.5	15095.0
**40**	USA	G1	+	30652.2	4620.7	406854.9	269223.3	10184.5	4040.0	25900.0	29479.0	2660.0	9719.0	70397.3
**41**	USA	G1	+	51885.2	23907.4	607640.5	179676.7	22906.4	5280.0	32400.0	51006.0	6980.0	16097.0	458976.1
**42**	Belgium	G4	pBLV344^e^	4.07E+10	4.15E+09	4E+09	7E+06	1.45E+09	2.9E+07	7.48E+09	2.27E+08	2.55E+11	2.59E+09	1.22E+11
**43**	USA	G1	BLV-FLK^f^	307692.3	162180.6	863440.0	3250666.7	75773.5	28480.0	168000.0	244769.0	482000.0	138382.8	4286730.3
**44**	Poland	G8	+	1.7	41.8	NEG	763.3	10.4	NEG	NEG	5.0	0.2	2.0	5.9
**Total**		**POSITIVE**	**43**	**43**	**35**	**37**	**36**	**40**	**32**	**40**	**42**	**42**	**42**	**41**
		**NEGATIVE**	**1**	**1**	**9**	**7**	**8**	**4**	**12**	**4**	**2**	**2**	**2**	**3**

^a^*NEG* negative result, ^b^*Ns* not sequenced, ^c^*Na* not applicable, ^d^in the qPCR two primer sets for *pol* gene MRBLVL and MRBLVR were used, ^e^pure plasmid DNA BLV344 (gift from Luc Willems), ^f^DNA isolated from Fetal Lamb Kidney cell line persistently infected with BLV (FLK-BLV), qPCR1- Auburn University College of Veterinary Medicine, US, qPCR2 CentralStar Cooperative, Michigan, US, qPCR3 - Laboratório Federal de Defesa Agropecuária de Minas Gerais (LFDA-MG), Brasil; qPCR4 - Centro de Investigación Veterinaria de Tandil (CIVETAN), Argentina; qPCR5 - Faculty of Agriculture Iwate University, Japan; ddPCR6 - Universidad de la República de Uruguay (UdelaR), Montevideo, Urugway; qPCR7 - Croatian Veterinary Institute, Zagreb, Croatia ; qPCR8 - Instituto Nacional de Tecnología Agropecuaria (INTA), Argentina; qPCR9 - Laboratorio Central de Veterinaria LCV Algete, Spain; qPCR10 - National Veterinary Institute (NVRI), Puławy, Poland; qPCR11 - French Agency for Food, Environmental and Occupational Health and Safety (Anses), Niort, France

Except for the positive (pBLV344 and FLK cell line) and the negative controls, all samples had previously shown detectable levels of BLV-specific antibodies (BLV-Abs) by enzyme-linked immunosorbent assays (ELISA). During the current interlaboratory study, both the positive and negative controls were assessed adequately by all eleven PCR tests. Of all 43 positive samples, 43, 35, 37, 36, 40, 32, 40, 42, 42, 42 and 41 samples were detected as positive by the qPCR1, qPCR2, qPCR3, qPCR4, qPCR5, ddPCR6, qPCR7, qPCR8, qPCR9, qPCR10 and qPCR11 methods, respectively. Based on these observations, the most sensitive method was the qPCR1, and the method with the lowest sensitivity was the ddPCR6. Twenty-nine out of 44 samples were identified correctly by all qPCRs. The remaining 15 samples gave discordant results. Comparison of qualitative results (positive versus negative) from all eleven methods revealed 87.33% overall agreement and a kappa value of 0.396 (Cohen's kappa method adapted by Fleiss) [44, 45]. The levels of agreement among the results from the eleven methods are represented in Table 3. The maximum agreement was seen between two methods (qPCR9 and qPCR10 [100% agreement and a Cohen's kappa value of 1.000]) that used similar protocols and targeted the same region of BLV *pol*.

**Table 3:** Assessment of qPCR results by pairwise comparison

PCR assay	Kappa value or agreement^a^ with the results of the following PCR assay										
	**qPCR1**	**qPCR2**	**qPCR3**	**qPCR4**	**qPCR5**	**ddPCR6**	**qPCR7**	**qPCR8**	**qPCR9**	**qPCR10**	**qPCR11**
qPCR1	-	0.166	0.219	0.189	0.377	0.117	0.377	0.656	0.656	0.656	0.482
qPCR2	81.8%	-	0.391	0.927	0.560	0.441	0.384	0.313	0.313	0.312	0.443
qPCR3	86.4%	81.8%	-	0.438	0,075	0.539	0.486	0.163	0.402	0.402	0.115
qPCR4	84.1%	97.7%	84.1%	-	0.431	0.488	0.431	0.137	0.353	0.352	0.495
qPCR5	93.2%	88.6%	79.5%	86.4%	-	0.276	0.450	0.645	0.290	0.290	0.845
ddPCR6	75.0%	79.6%	84.1%	81.8%	77.3%	-	0.421	0.070	0.225	0.225	0.326
qPCR7	93.2%	84.1%	88.6%	86.4%	90.9%	81.8%	-	0.290	0.290	0.290	0.535
qPCR8	97.7%	84.1%	84.1%	66.8%	95.5%	72.7%	90.9%	-	0.476	0.476	0.365
qPCR9	97.7%	84.1%	88.6%	86.4%	90.9%	77.3%	90.9%	95.5%	-	1.00	0.365
qPCR10	97.7%	84.1%	88.6%	86.4%	90.9%	77.3%	90.9%	95.5%	100%	-	0.365
qPCR11	95.5%	86.4%	81.8%	88.6%	97.7%	79.6%	93.2%	93.2%	93.2%	93.2%	-

^a^Values above the blank cells are kappa values; values below the blank cells are percentages of agreement

### Analysis of BLV pol, env and LTR sequences targeted by particular PCR assays

**Fig. 2:**
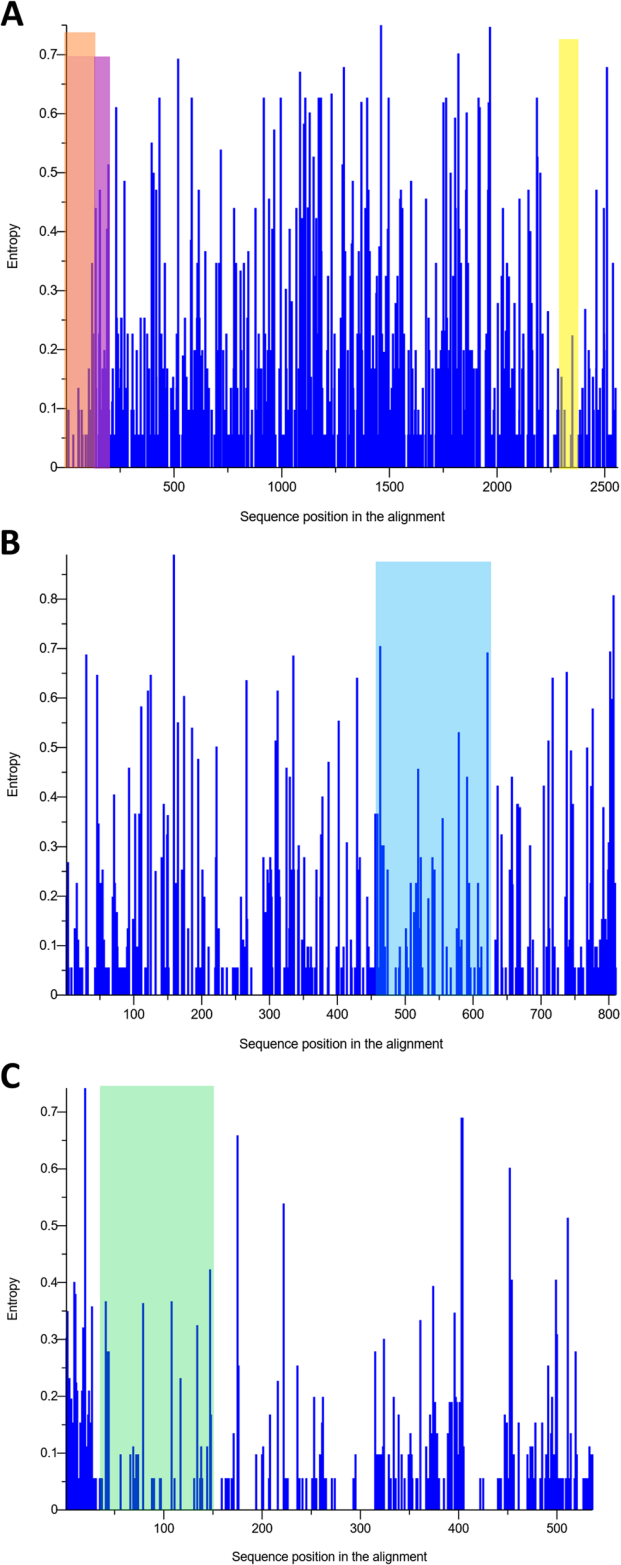
Sequence variability measured as per-site entropy. **A** Multiple alignment of the *pol* gene showing the locations of qPCR fragments in regions of the *pol* gene for the qPCR1 (highlighted in pink), qPCR4 (highlighted in yellow) and for the qPCR7, qPCR8, qPCR9, qPCR10 and qPCR11 assays (highlighted in orange). **B** Multiple alignment of the *env* gene targeted by ddPCR6 (highlighted by blue rectangle). **C** Multiple alignment of the LTR region by qPCR3 (highlighted in mint)

Due to differences in performance observed among the *pol*-based qPCR assays (the qPCR1, qPCR2, qPCR4, qPCR5 and qPCR7- qPCR11 methods), and considering that the *env*-based ddPCR6 and LTR-based qPCR3 assay showed the lowest sensitivity and the poorest agreement with the other assays, the degree of sequence variability between the *pol*, *env* and LTR genes was addressed. From the MSAs for *pol*, *env* and LTR, the nucleotide diversity (π) was calculated. The π value for *pol* gene was lower than that for LTR and *env* gene (π_*pol*_, 0.023 [standard deviation {SD}, 0.018]; π_LTR_, 0.024 [SD, 0.011]; π_*env*_, 0.037 [SD, 0.013]). From this analysis, *pol* sequences appeared to be less variable than *env* and LTR sequences. In addition, we performed a Shannon entropy-based per-site variability profile of the *pol*, *env* and LTR sequences used in this study (Fig. 2A-C).

The all-observed entropy plots were homogeneous along the whole sequences. Considering the three regions of *pol* gene, the highest entropy (4.67) occurred in the region targeted by the qPCR1 primers, whereas the entropy for qPCR7—qPCR11 and qPCR4 primers were 1.57 and 0.38, respectively. For the LTR region targeted by qPCR3 primers and for *env* gene targeted by ddPCR6, the total entropy was equal to 4.46 and 7.85, respectively. This analysis showed a marked region of variability for LTR and *env* fragments. Interestingly, we noted that the qPCR7—qPCR11 targeted the most conserved regions of reverse transcriptase and qPCR4 primers targeted the most-conserved region of virus integrase (Fig. 2A-C; see also Additional file 7).

### Quantitation of BLV proviral DNA by different qPCR/ddPCR assays

**Fig. 3:**
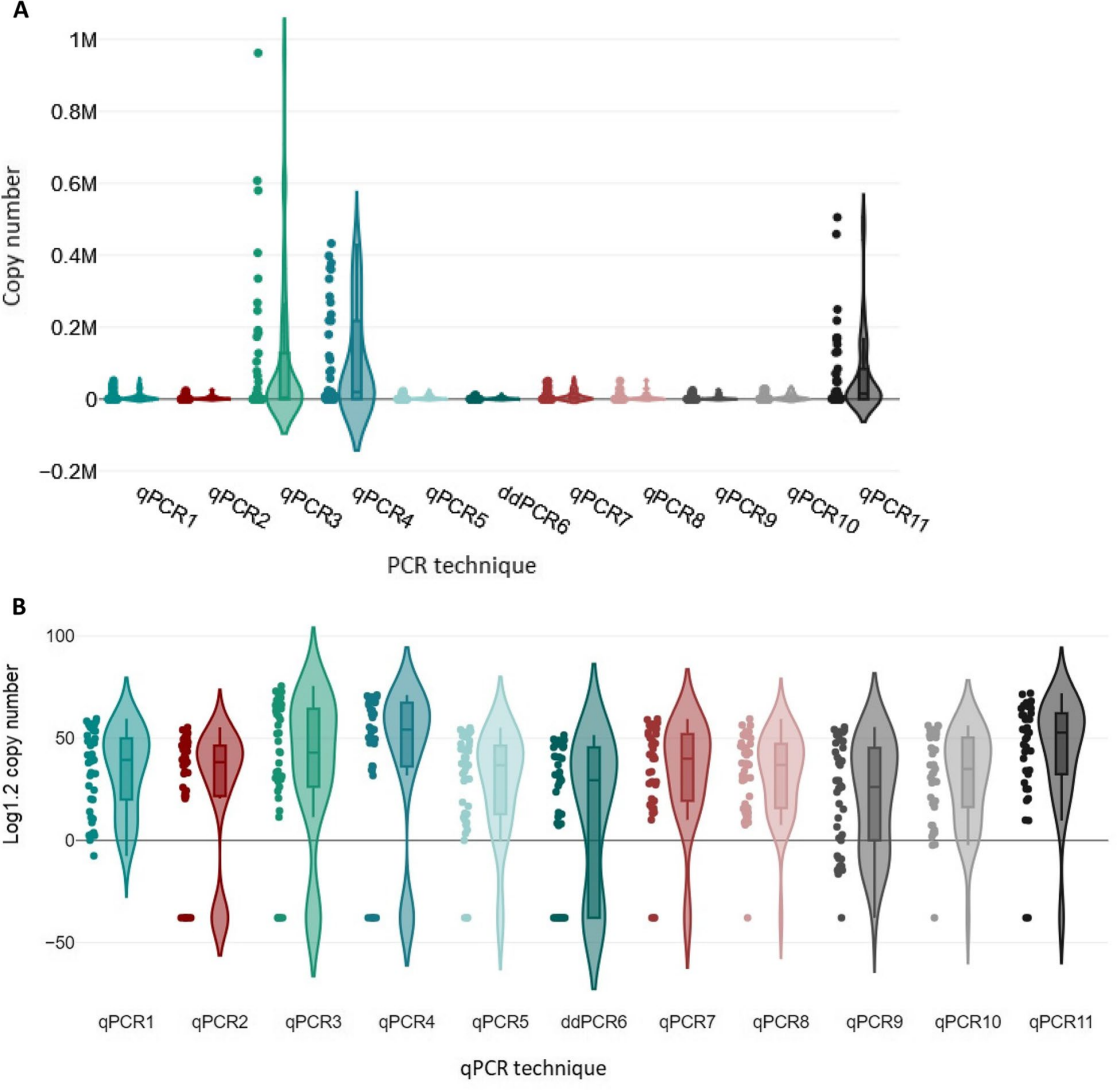
Comparison of detection of BLV proviral DNA copy numbers by eleven testing methods. Shown is a box plot of data from Kruskal–Wallis ANOVA, a rank test. The DNA copy numbers for 41 samples, determined independently by each of the 11 qPCRs, were used for the variance analysis. In this analysis, the positive controls (sample ID 42 and ID 43) and negative control (sample ID 32) were excluded. **A** Violin plot for graphical presentation of the ANOVA of proviral copy number values. **B** Violin plot for ANOVA analysis of variance, copy number values are presented on a logarithmic scale (Log1.2) for better illustration of copy number differences between PCR methods

To analyze whether the range of copy numbers detected by each qPCR was comparable to those of the others, Kruskal–Wallis one-way analysis of variance (ANOVA) was used. The violin plots were used to visualize the ANOVA results (Fig. 3A-B).

**Fig. 4:**
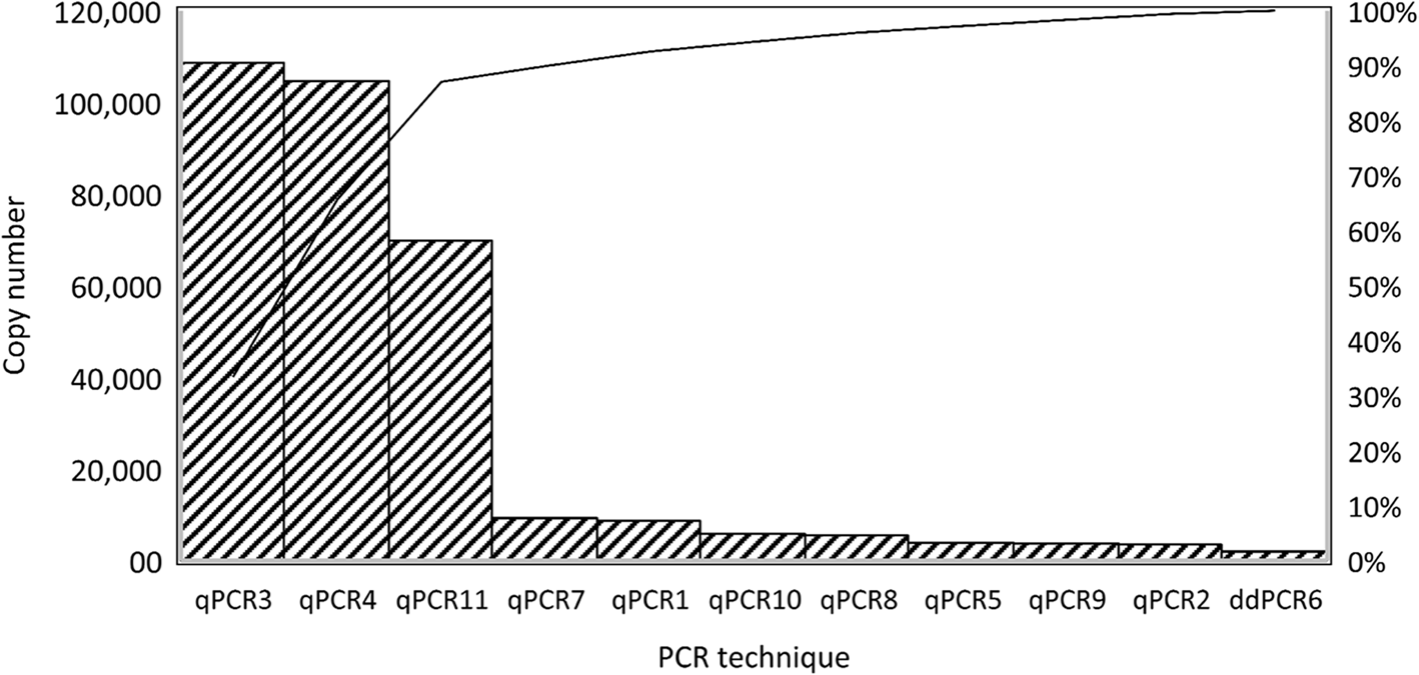
A Pareto chart with the proviral BLV copy mean values for eleven PCR assay arranged in descending order. Pareto charts was prepared based on 80–20 rule, which states that 80% of effects come from 20% of the various causes

The grouping variable revealed significant differences among the distributions of proviral DNA copy numbers with the various qPCRs (*P* < 0.001). These results showed that the abilities of qPCRs/ddPCR to determine the proviral DNA copy number differed. A Dunn-Bonferroni test was used to compare the groups in pairs to find out which was significantly different. The Dunn-Bonferroni test revealed that the pairwise group comparisons of qPCR2—qPCR4, qPCR3—ddPCR6, qPCR4—qPCR5, qPCR4—ddPCR6, qPCR4—qPCR9, qPCR4—qPCR10, qPCR5—qPCR11, ddPCR6—qPCR11 and qPCR9—qPCR11 have an adjusted P value less than 0.05 and thus, it can be assumed that these groups were significantly different in each pair (see Additional file 4). The Pareto chart was used to show the average copy number values of all methods in descending order. These Pareto charts were prepared based on 80–20 rule, which states that 80% of effects come from 20% of the various causes [46]. The methods that generated the highest copy numbers was qPCR3 and qPCR4, on the other hand the lowest copy numbers and/or highest negative results were generated by ddPCR6 (Fig. 4).

**Fig. 5:**
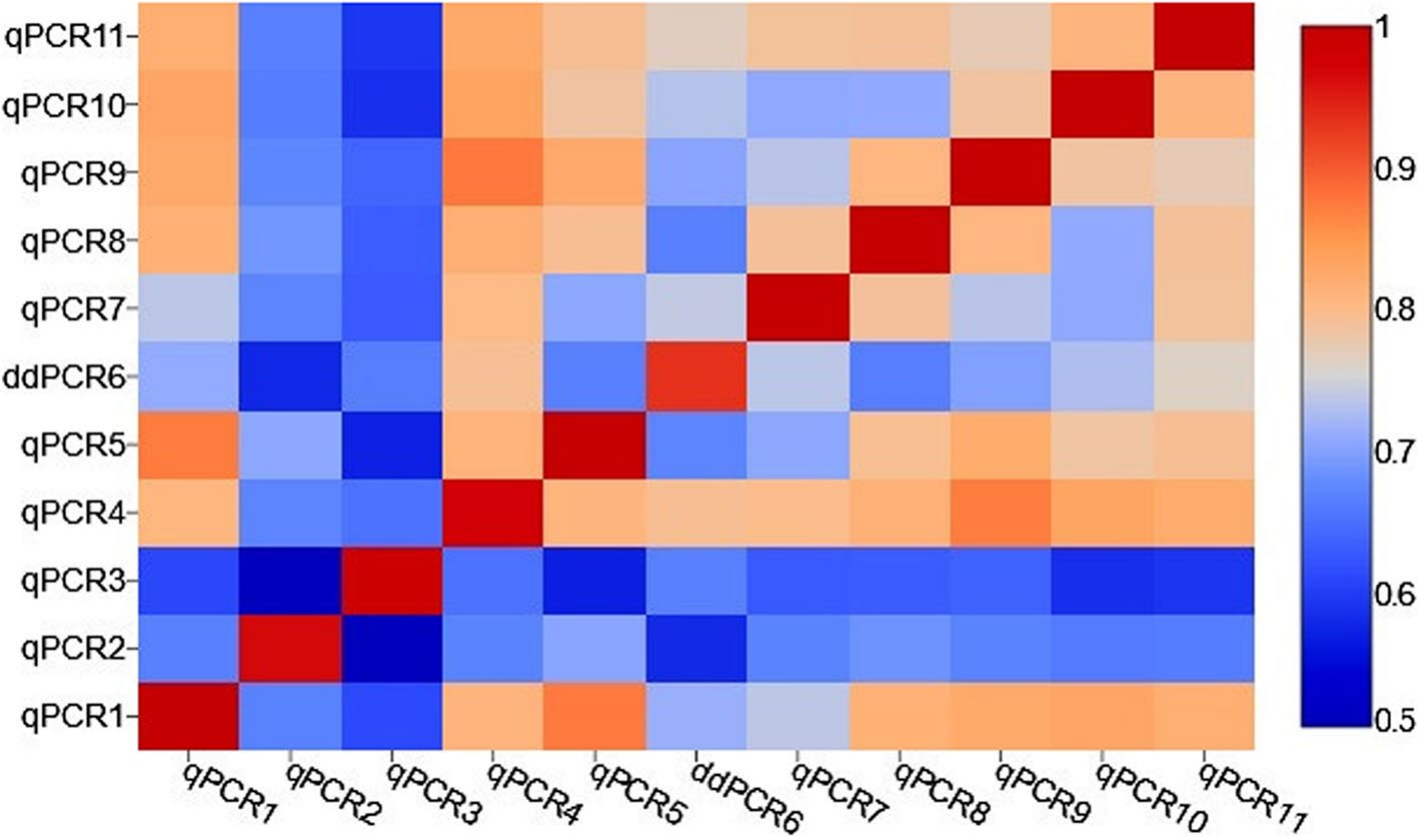
The heatmap of Kendall’s tau correlation coefficients between copy numbers detected by ten qPCRs and one ddPCR. Statistically significant differences in the distribution of copy numbers, a moderate, strong and very strong correlation between particular qPCRs/ddPCR was observed. The strength of the association, for absolute values of r, 0–0.19 is regarded as very weak, 0.2–0.39 as weak, 0.40–0.59 as moderate, 0.6–0.79 as strong and 0.8–1 as very strong correlation

The correlations between copy numbers detected by different qPCRs and ddPCR assays were calculated. The Kendall's Tau correlation coefficient measured between each pair of the assays was shown in the Additional file 5 and in Fig. 5 as a correlation heatmap. The average correlation for all qPCRs and ddPCR assays was strong (Kendall's tau = 0.748; *P* < 0.001).

**Fig. 6:**
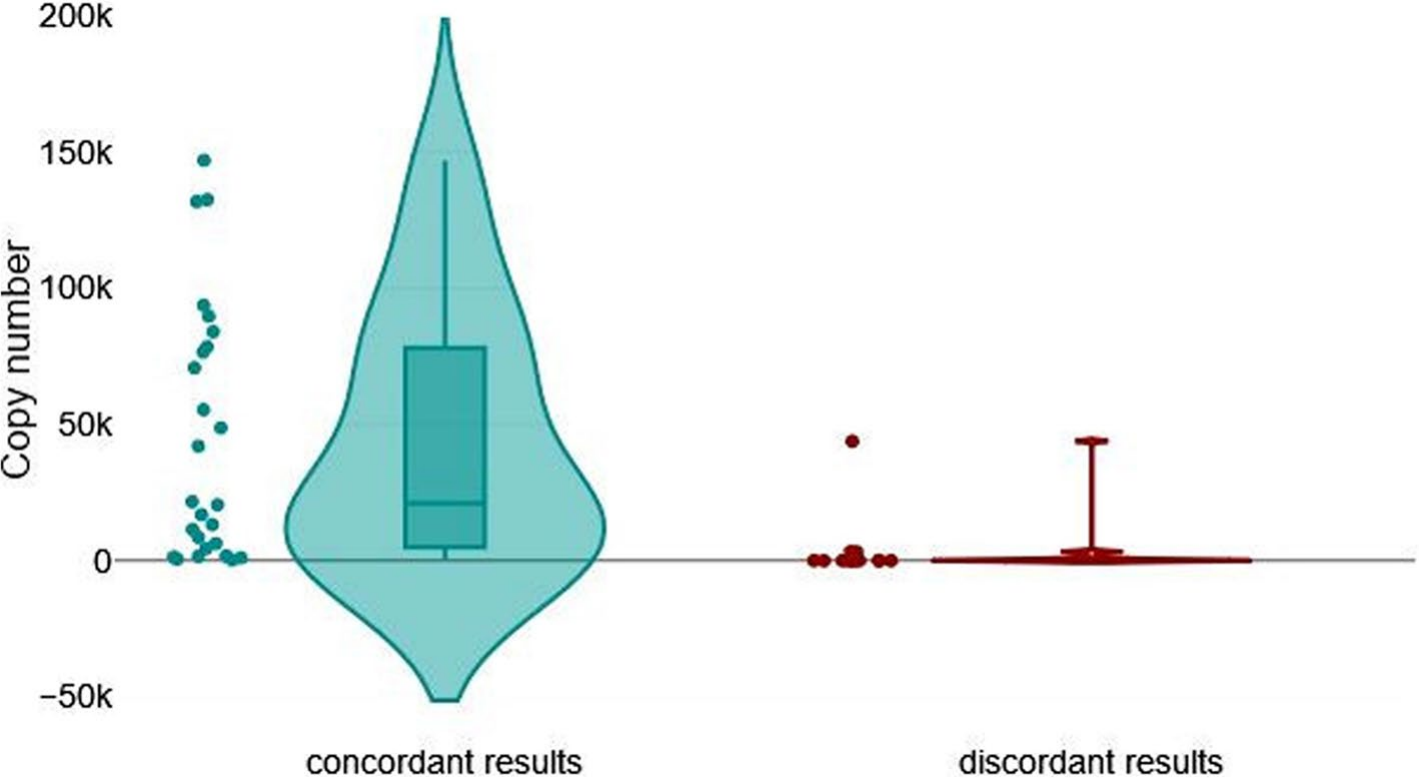
Impact of BLV proviral copy numbers on the level of agreement. Violin plot for graphical presentation of Mann–Whitney U test. The test was performed to compare BLV provirus copy number in two groups of samples: 28 samples with fully concordant results from all eleven qPCR/ddPCR assays (left) and 15 samples with discordant results from different qPCR/ddPCR assays (right) (*P* < 0.001). Sample ID 42 was excluded from the statistical analysis

Since the differences between PCR tests may be influenced by the number of BLV proviral copies present in each sample, we compared the average number of BLV copies between a group of genomic DNA samples that gave concordant results (group I [*n* = 28]) and a group that gave discordant results (group II [*n* = 15]). The mean number of copies was 73,907 (minimum, 0; maximum, 4,286,730) in group I, and 3,479 (minimum, 0; maximum, 218,583) in group II, and this difference was statistically significant (*P* < 0.001 by a Mann–Whitney U- test) (Fig. 6).

The results show that the concordant results group had considerably higher copy numbers (median, 5,549.0) than the discordant results group (median, 6.3).

## Discussion

BLV control and eradication programs consist of correct identification and subsequent segregation/elimination of BLV-infected animals [47]. Detection of BLV- infected cows by testing for BLV-specific antibodies in serum by agar gel immunodiffusion and ELISA is the key step and standard to be implemented of EBL eradication programs according to WOAH (https://www.woah.org/en/disease/enzootic-bovine-leukosis/) [9]. Despite the low cost and high throughput of serological tests, there are several scenarios where highly specific and sensitive molecular assays for the detection of BLV DNA might improve detection and program efficiency.

In this perspective, qPCR assays can detect small quantities of proviral DNA during acute infection, in which animals show very low levels of anti-BLV antibodies [43, 48–50]. qPCR methods can also work as confirmatory tests to clarify ambiguous and inconsistent serological test results [11]. Such quantitative features of qPCRs are crucial when eradication programs progress and prevalence decreases. Moreover, qPCR allows not only the detection of BLV infection but also estimation of the BLV PVL, which directly correlates with the risk of disease transmission [51, 52]. This feature of qPCR allows for a rational segregation of animals based on the stratified risk of transmission. These considerations allow for greater precision in the management of BLV within large herds with a high prevalence of BLV ELISA-positive animals to effectively reduce herd prevalence [13, 53]. BLV is a global burden and the lack of technical standardization of molecular detection systems remains a huge obstacle to compare surveillance data globally based on the first interlaboratory trial performed in 2018 [15]. In the 2018 study we observed an adjusted level of agreement of 70% comparing qualitative qPCR results; however, inconsistencies amongst methods were larger when low number of copies of BLV DNA were compared. Samples with low copies of BLV DNA (< 20 copies per 100 ng) accounted for the higher variability and discrepancies amongst tests. We concluded from the first interlaboratory trial that standardizing protocols to improve sensitivity of assays with lower detection rates was necessary.

In this follow up study, we re-tested the TaqMan BLV qPCR developed and validated by NVRI (acting as reference WOAH laboratory) and the one adapted from this original protocol to be used with SYBR Green dye, allowing a significant reduction in costs [11]. Another 3 laboratories also performed NVRI´s qPCR with slight modifications (i.e., Spain performed a multiplex assay for internal normalization). The remaining 6 labs introduced novel methodologies to the trial including one ddPCR (UY).

To compare different qPCR methods, a more comprehensive sample panel, accounting for a more geographical diversification was used in this trial. The amounts of BLV DNA in these samples were representative of the different BLV proviral loads found in field samples (from 1 to > 10,000 copies of BLV proviral DNA). Of note, 34% of reference samples had less than 100 copies of BLV DNA per 100 ng; samples were lyophilized to grant better preservation and reduced variability during distribution to participants around the globe.

The panel included a single negative control and two positive controls. Diagnostic sensitivity (DxSn) was estimated for each qPCR. Considering the 43 positive samples, the DxSn for the different qPCRs were: qPCR1 = 100%, qPCR2 = 82%, qPCR3 = 86%, qPCR4 = 84%, qPCR5 = 93%, ddPCR6 = 74%, qPCR7 = 93%, qPCR8 = 98%, qPCR9 = 98%, qPCR10 = 98% and qPCR11 = 95%. The most sensitive method was the qPCR1, and the method with the lowest sensitivity was the ddPCR6 method. Twenty-nine out of 44 samples were identified correctly by all qPCRs. The remaining 15 samples gave discordant results. The comparison of qualitative qPCR results among all raters revealed an overall observed agreement of 87%, indicating strong interrater reliability (Cohen´s kappa = 0.396) [54, 55].

There are several factors that contribute to variability in qPCR results (i.e., number of copies of target input, sample acquisition, processing, storage and shipping, DNA purification, target selection, assay design, calibrator, data analysis, etc.). For that reason and as expected, the level of agreement among sister qPCRs (qPCR7, qPCR9-11) sharing similar protocols was higher compared to the rest of assays; this was also true for qPCR8 which targets the same region of BLV *pol* gene (shares same primers) but has a particular set-up to be used with SYBR Green chemistry. Oppositely, lower sensitivity and larger discrepancy against other tests was observed for the ddPCR6 and qPCR2-4.

Based on these observations we investigated which factors might have accounted for larger assessment variability amongst tests. In the first place, we observed that the use of different chemistries was not detrimental for the sensitivity and agreement among tests; similar DxSn and comparable level of agreement were obtained comparing TaqMan (qPCR7, 10, 11) vs SYBR Green (qPCR8) chemistries while targeting identical BLV sequence and using same standards. Also, when a multiplex qPCR (TaqMan) targeting the same BLV sequence and using the same standard was compared to previous ones, agreement was kept high, indicating that the lower sensitivity described for some multiplex qPCRs did not take place in this comparison. The use of an international calibrator and the efficiency estimation (standard curve) might inform variability associated with different chemistries. In contrast, another multiplex assay targeting another region of BLV *pol* (qPCR2) showed much lower sensitivity and agreement. As qPCR2 is performed as service by private company and oligonucleotide sequences were not available, we were not able to investigate in which proportion each of these two variables contributed to the lower performance of this assay, but we note the addition of 4 µl genomic DNA to this assay that would have an impact the DxSn. In this regard, there is substantial evidence showing that the variability of target sequence among strains from different geographical areas, might affect the sensitivity of BLV qPCRs. Previous studies comparing the *pol*, *gag*, *tax* and *env* genes reported that the *pol* gene was the most suitable region to target for diagnostic purposes, since it provided the most-sensitive assays [11, 15, 56–59]. This might be due in part to higher sequence conservation of *pol* among strains from different geographical areas. Supporting this observation, it is noticeable how JPN qPCR improved their performance in the current trial, by targeting *pol* in place of *tax*, as it did in the previous interlaboratory trial. Since it is a commercial test, we cannot exclude other factors contributing for the performance upgrade observed for this qPCR. In the current study, qPCR3 and ddPCR6 targeting LTR and *env* sequences, showed lower performances than other assays. Standardization of DNA input into each qPCR would have likely resulted in higher concordance in results. For instance, qPCR1 added 10 µl of genomic DNA per reaction and ddPCR6 added 1 µl of genomic DNA, impacting the resulting sensitivity differences.

Since the sensitivity of each assay and, consequently, the level of agreement among assays might also be influenced by the number of BLV DNA copies present in each sample [48], we compared the average number of BLV DNA copies between a group of genomic DNA samples that gave concordant results and a group that gave discordant results, and observed that samples that gave discordant results had significantly lower numbers of BLV DNA copies than samples that gave concordant results. Related to this point, the degradation of target DNA during lyophilization, shipment and resuspension, could have been more significant in low-copy compared to high-copy samples. Consequently, the degradation of target DNA in samples with low copies of BLV DNA might have accounted for the greater level of discrepancy within this subset of samples. The rational of adding a large proportion of such samples (34% samples with less than 100 BLV copies per 100 ng of total DNA) was to mimic what is frequently observed in surveillance programs (i.e., hyperacute infection, chronic asymptomatic infection, etc.).

Quantitative methods for the detection of BLV DNA copies are important for segregation programs based on animal level of BLV PVL, as well as for scientific research and the study of BLV dynamics. When the numbers of copies of BLV DNA detected by different assays were compared, in the present study, we observed that although the ability to quantify BLV DNA differed among qPCRs/ddPCR and there were statistically significant differences in the distribution of copy numbers among assays, a strong average correlation was found for the eleven qPCRs/ddPCR. In this regard, the lack of an international calibrator (standard curve) could be a major contributor to the increment of quantitative variation amongst laboratories. For that reason, plasmid pBLV1 containing *pol* 120 bp sequence was originally constructed for use as standard for quantification and shared with some collaborators (i.e., qPCR7, qPCR8, qPCR 9, qPCR10 and qPCR11). Remarkably, the laboratories used pBLV1 standard in the current trial obtained the most comparable results, indicating that the use of an international standard may have significant impact on the convergence of results; such standard reference material should be prepared under identical conditions. To avoid further variability a detailed protocol for lyophilized DNA sample resuspension, quantitation and template input into each qPCR should be shared with all participants.

## Conclusions

BLV DNA was detected with different level of sensitivity in serologically positive samples from different origin and classified into different BLV genotypes. Overall agreement was high; however, we found significant differences in results for the samples with low BLV DNA copy numbers. This second interlaboratory study demonstrated that differences in target sequence, DNA input and calibration curve standards can increase interlaboratory variability considerably. Next steps should focus on (i) standard unification (international gold standard) to estimate individual test efficiency and improve quantitative accuracy amongst tests; (ii) building a new panel of samples with low BLV DNA copy numbers to re-evaluate sensitivity and quantitation of molecular methods. Since no variation was observed in samples from different genotypes, all samples will be collected in Poland to standardize the collection, purification, lyophilization and shipping steps with precise instructions for suspension and constant input volume for the PCR reaction. Finally, we believe that following this standardization approach we will be able to improve overall agreement amongst tests, improving the diagnostic of BLV around the world.

### Supplementary Information

Additional file 1. Copy of the instruction included with the panel of 44 DNA samples sent to participating laboratories for dilution of the lyophilisates

Additional file 2. Detection of the H3F3A gene copy number in 43 DNA samples; no outlier was found for any samples (*P* <0.05) (two-sided).

Additional file 3. Concentration values of 44 DNA samples measured by the 11 participating laboratories (given in ng per µl)

Additional file 4. Post hoc - Dunn-Bonferroni-Tests. The Dunn-Bonferroni test revealed that the pairwise group comparisons of qPCR2 - qPCR4, qPCR3 - ddPCR6, qPCR4 - qPCR5, qPCR4 - ddPCR6, qPCR4 - qPCR9, qPCR4 - qPCR10, qPCR5 - qPCR11, ddPCR6 - qPCR11 and qPCR9 - qPCR11 have an adjusted p-value less than 0,05

Additional file 5. Kendall's Tau correlation coefficient values measured between each pair of assays. The numbers 1 to 11 in the first column and last row of the table indicate the names of the assays qPCR1-qPCR5, ddPCR6, qPCR7-qPCR11 respectively

Additional file 6. Maximum-likelihood phylogenetic analysis of full-length BLV-pol gene sequences representing 7 BLV genotypes (G1, G2, G3, G4, G6, G9, and G10) (A); (B) env-based sequences assigned to 10 BLV genotypes (G1, G2, G3, G4, G5, G6, G7, G8, G9, and G10); (C) LTR-based sequences representing 10 BLV genotypes (G1-G10). For all genes and LTR region the Tamura-Nei model and Bootstrap replications (1,000) were applied in MEGA X

Additional file 7. Multiple sequence alignment of reverse transcriptase, integrase, envelope and LTR sequences in the context of the specific primers used by different qPCR assays. (A) Multiple sequence alignment of reverse transcriptase (pol gene) sequences in the context of qPCR7, qPCR8, qPCR9, qPCR10 and qPCR11 assay primers. (B) Multiple sequence alignment of integrase (pol gene) sequences in the context of qPCR4 assay primers. (C) Multiple sequence alignment of env gene sequences in the context of ddPCR6. (D) Sequence alignment of LTR region sequences in the context of qPCR3 method primers

## Data Availability

Not applicable.

## Abbreviations

ANOVA: One-way analysis of variance
BLV: Bovine leukemia virus
BLV-Abs: BLV-specific antibodies
ddPCR: Digital PCR
DxSn: Diagnostic sensitivity
EBL: Enzootic bovine leukosis
ELISA: Enzyme-linked immunosorbent assays
FRET: Real-time fluorescence resonance energy transfer PCR
GQN: Genomic quality number
H3F3A: Histone H3 family 3A housekeeping gene
ML: Maximum likelihood phylogenetic tree
MSA: Multiple-sequence alignment
PBLs: Peripheral blood leukocytes
PBS: Phosphate-buffered saline
PVL: Proviral load
qPCR: Quantitative real-time PCR
RT: Room temperature
WOAH: World Organisation for Animal Health

## Data availability

No datasets were generated or analysed during the current study.
