# Supplementary material for: Inter-laboratory comparison of eleven quantitative or digital PCR assays for detection of proviral bovine leukemia virus in blood samples
Source: BMC Vet Res. 2024 Aug 26;20:381. doi: 10.1186/s12917-024-04228-z (PMC11346035; doi:10.1186/s12917-024-04228-z)
Supplement: Supplementary file 1 — Additional file 1. Copy of the instruction included with the panel of 44 DNA samples sent to participating laboratories for dilution of the lyophilisates [file 12917_2024_4228_MOESM1_ESM.pdf]

NATIONAL VETERINARY RESEARCH INSTITUTE
Department of Biochemistry
National Reference Laboratory for Enzootic Bovine Leukosis
Al. Partyzantów 57 24-100 Puławy
tel. 081 889 30 00 fax. 081 886 25 95

PIWet

OiE
Reference Laboratory for
Enzootic Bovine Leukosis

August 11, 2022

Dear Participants, dear colleagues,

I prepared a panel of 44 DNA samples to perform interlaboratory comparison study on BLV detection using real time PCR. Genomic DNA was extracted from peripheral blood leukocytes or whole blood of cattle naturally infected with BLV and the samples were collected from cows from different countries: Poland, Moldova, Ukraine, Pakistan, Canada and USA. Enclosed, please find the panel of these samples containing **lyophilized DNA**.
The parcels will be sent by a regular mail. I will appreciate very much if you could dissolve the lyophilisates in PCR-clean water, measure the DNA concentration, and test these samples using your qPCR, available in your laboratory, estimate the provirus copy number in **100 ng** of genomic DNA and send me back the results to the correspondence address: aneta.pluta@piwet.pulawy.pl

INSTRUCTION TO DISSOLVE LYOPHILIZED DNA SAMPLES:

1. Add 20-25 µl of PCR-clean water to each sample.
2. Do not pipette.
3. Incubate the samples for 45 min at room temperature.
4. During this time, set the heater block to 55°Celsius.
5. Then transfer the samples and incubate for 5 min at 55°C.
6. Remove samples and gently vortex for 10 seconds.
7. Gently spin each sample.
8. Measure the concentration.

The concentrations of the samples should range from 40-200 ng /ul.

NOTE: Be careful not to contaminate each other when dissolving the samples in water as the panel contains both highly positive, low positive and BLV negative samples.

IMPORTANT INFORMATION:
Submitted samples cannot be used for other purposes not related to interlab assay.

Thank you in advance for your collaboration.

Aneta Pluta, PhD
Assistant Professor, Department of Biochemistry
Department of Omics Analysis
National Veterinary Research Institute, Poland
Av. Partyzantów 57, 24-100 Puławy
tel. 48 81 889 3113/3375
aneta.pluta@piwet.pulawy.pl

PAŃSTWOWY INSTYTUT WETERYNARYJNY-
PAŃSTWOWY INSTYTUT BADAWCZY
Zakład Biochemii
Al. Partyzantów 57, 24-100 Puławy
Tel. (081) 889-30-00, (081) 889-31-11
Fax (081) 886-25-95

11.08.2022r.
Pluta Aneta
