# Supplementary figures and images for: Inter-laboratory comparison of eleven quantitative or digital PCR assays for detection of proviral bovine leukemia virus in blood samples
Source: BMC Vet Res. 2024 Aug 26;20:381. doi: 10.1186/s12917-024-04228-z (PMC11346035; doi:10.1186/s12917-024-04228-z)

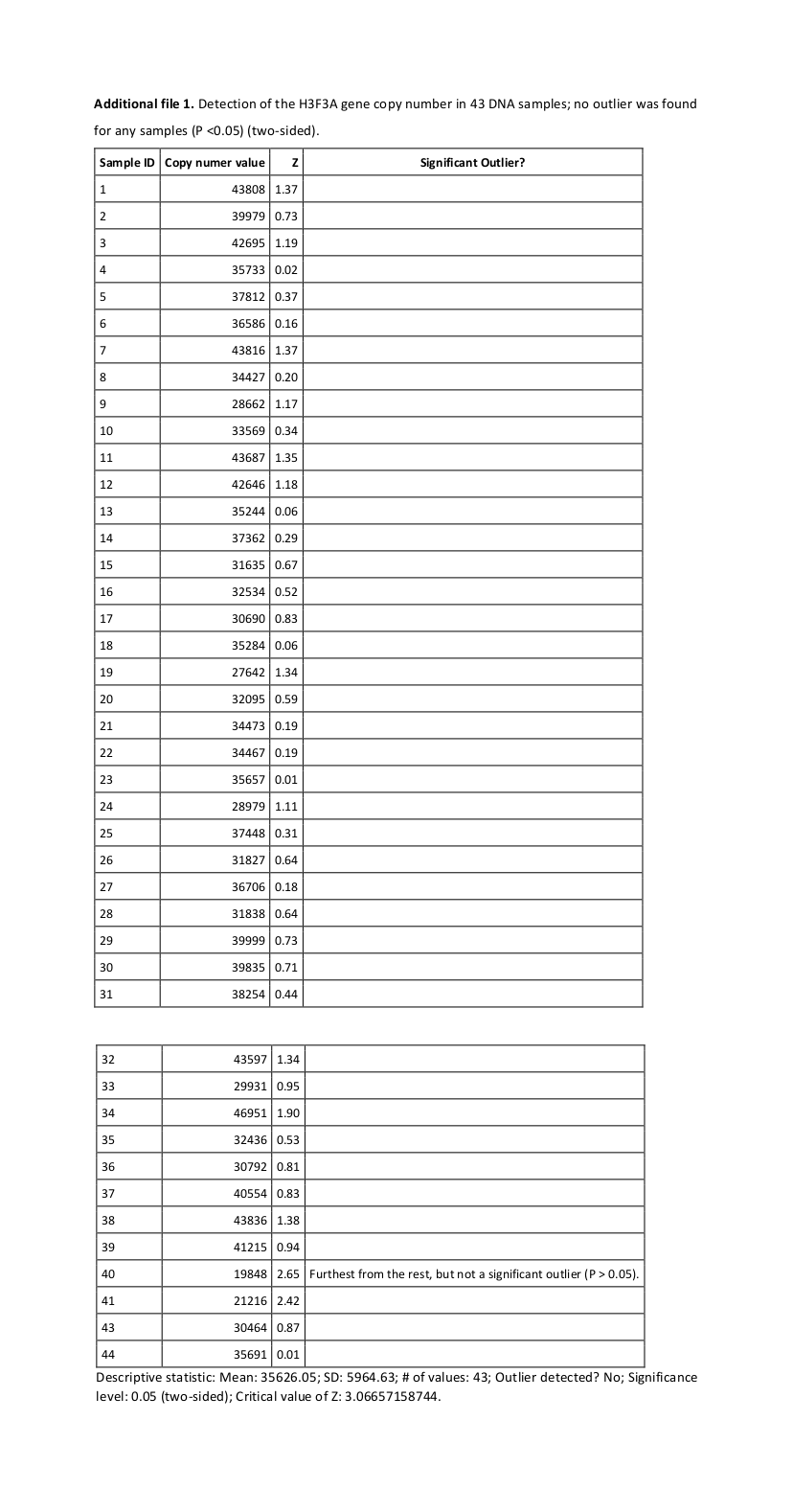

Supplement: Supplementary file 2 — Additional file 2. Detection of the H3F3A gene copy number in 43 DNA samples; no outlier was found for any samples (P <0.05) (two-sided). [file 12917_2024_4228_MOESM2_ESM.png]

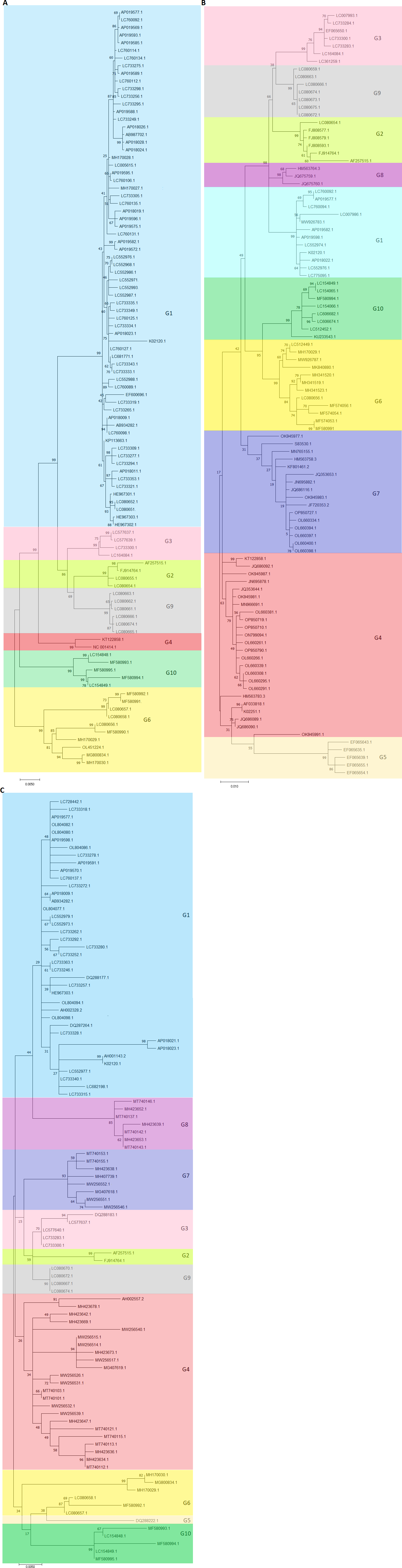

Supplement: Supplementary file 6 — Additional file 6. Maximum-likelihood phylogenetic analysis of full-length BLV-pol gene sequences representing 7 BLV genotypes (G1, G2, G3, G4, G6, G9, and G10) (A); (B) env-based sequences assigned to 10 BLV genotypes (G1, G2, G3, G4, G5, G6, G7, G8, G9, and G10); (C) LTR-based sequences representing 10 BLV genotypes (G1-G10). For all genes and LTR region the Tamura-Nei model and Bootstrap replications (1,000) were applied in MEGA X [file 12917_2024_4228_MOESM6_ESM.png]
