# Supplementary material for: Inter-laboratory comparison of eleven quantitative or digital PCR assays for detection of proviral bovine leukemia virus in blood samples
Source: BMC Vet Res. 2024 Aug 26;20:381. doi: 10.1186/s12917-024-04228-z (PMC11346035; doi:10.1186/s12917-024-04228-z)
Supplement: Supplementary file 3 — Additional file 3. Concentration values of 44 DNA samples measured by the 11 participating laboratories (given in ng per µl) [file 12917_2024_4228_MOESM3_ESM.docx]

Additional file 3. Concentration values of 44 DNA samples measured by the 11 participating laboratories (given in ng per µl).

|  | | | | |  | Nucleic Acid Conc. (ng/ul) | | |  |  |  |
| --- | --- | --- | --- | --- | --- | --- | --- | --- | --- | --- | --- |
| Sample No. | qPCR1 | qPCR2 | qPCR3 | qPCR4 | qPCR5 | ddPCR6 | qPCR7 | qPCR8 | qPCR9 | qPCR10 | qPCR11 |
| 1 | 96.5 | 46.4 | 47.2 | 72.1 | 80.7 | 71.4 | 55.6 | 70 | 86.6 | 58.0 | 122 |
| 2 | 78.7 | 84 | 60.5 | 97.2 | 56.3 | 76.5 | 40.3 | 67 | 73.8 | 29.5 | 59.6 |
| 3 | 82 | 52.8 | 47.5 | 52.6 | 58.9 | 57.2 | 48 | 57 | 83.2 | 41.5 | 78 |
| 4 | 84 | 35.7 | 57.2 | 48.9 | 61.7 | 63.4 | 120 | 63 | 68 | 50.5 | 55 |
| 5 | 88.3 | 30.2 | 57.6 | 45.5 | 53.8 | 58.8 | 121 | 48 | 75.9 | 58.5 | 3.3 |
| 6 | 104 | 13.4 | 53.5 | 50.7 | 45.6 | 40.3 | 126 | 65 | 94.7 | 45.5 | 63.2 |
| 7 | 90.3 | 47.2 | 66.4 | 67.3 | 63.7 | 79 | 137 | 67 | 82 | 49.0 | 63.2 |
| 8 | 143 | 105 | 61.2 | 133.4 | 131.5 | 111.9 | 194 | 125 | 116.6 | 88.7 | 85.4 |
| 9 | 86 | 47.6 | 63.1 | 85.7 | 89 | 80.3 | 81 | 69 | 6.9 | 79.0 | 54.8 |
| 10 | 86 | 43.7 | 62.8 | 86.9 | 117.3 | 110.9 | 110 | 76 | 69.5 | 76.7 | 58 |
| 11 | 118.5 | 42.4 | 63 | 111.4 | 121 | 120.1 | 161 | 91 | 94.5 | 94.7 | 88 |
| 12 | 98.5 | 38.1 | 43.9 | 90.2 | 94.8 | 91.2 | 117 | 80 | 86.5 | 70.5 | 61.8 |
| 13 | 43.6 | 62.8 | 35.4 | 57.6 | 61.7 | 60.1 | 66 | 49 | 48.3 | 28.3 | 28 |
| 14 | 99.2 | 57.3 | 25.7 | 101.4 | 109.5 | 143.2 | 104 | 119 | 84.4 | 91.3 | 69.4 |
| 15 | 75.4 | 39 | 53 | 76.9 | 81.3 | 83.5 | 69 | 83 | 65.6 | 50.7 | 53.2 |
| 16 | 83.6 | 53.7 | 55 | 86.4 | 94 | 124.3 | 69 | 75 | 75.2 | 76.0 | 66 |
| 17 | 120 | 85 | 77 | 125.9 | 181.1 | 129.1 | 222 | 98 | 104.4 | 105.0 | 98.6 |
| 18 | 46.7 | 30.4 | 41.6 | 54.9 | 55.9 | 55.4 | 38.3 | 49 | 66.8 | 106.0 | 41.2 |
| 19 | 97 | 123 | 82.3 | 105.1 | 112.9 | 107.3 | 82 | 92 | 83.3 | 82.7 | 69.4 |
| 20 | 69 | 53.6 | 56.3 | 75.4 | 81.7 | 83.1 | 79.6 | 72 | 67.7 | 60.3 | 56.6 |
| 21 | 92.6 | 29.3 | 73.4 | 95.6 | 101.2 | 91.7 | 84 | 76 | 77 | 68.0 | 67 |
| 22 | 178 | 153 | 147 | 26.6 | 267.1 | 212.8 | 220 | 217 | 158.8 | 168.0 | 119 |
| 23 | 112 | 43.6 | 94.4 | 89.6 | 94 | 112 | 122 | 76 | 98.7 | 78.0 | 71.4 |
| 24 | 55 | 27.1 | 28.8 | 49.2 | 51.1 | 56.7 | 43 | 60 | 39 | 36.3 | 42.6 |
| 25 | 58.5 | 34 | 37.5 | 55.1 | 54.1 | 154 | 31 | 87 | 49 | 31.5 | 41.6 |
| 26 | 72 | 24.7 | 18.8 | 58.9 | 75.8 | 58.3 | 34 | 74 | 47.2 | 21.5 | 47.2 |
| 27 | 90 | 34.4 | 36.6 | 117.3 | 105.1 | 105 | 160 | 119 | 86.7 | 82.7 | 74 |
| 28 | 85 | 46.7 | 64.4 | 90.1 | 86.5 | 86.3 | 165 | 64 | 69.9 | 64.7 | 58.8 |
| 29 | 115 | 44 | 35.5 | 140.2 | 148.5 | 137 | 161 | 200 | 110.8 | 104.0 | 102 |
| 30 | 98 | 45 | 46.1 | 84.9 | 105.9 | 83.3 | 131 | 70 | 69.8 | 84.0 | 62.4 |
| 31 | 44 | 16.4 | 24.4 | 43.6 | 45.9 | 49.3 | 38 | 48 | 34.5 | 28.1 | 27 |
| 32 | 67 | 18.8 | 29 | 60.5 | 73 | 62 | 35.7 | 90 | 39.9 | 178.0 | 36.6 |
| 33 | 34 | 21.3 | 23.1 | 34.1 | 36.7 | 39.5 | 44.4 | 44 | 27.7 | 23.8 | 21.2 |
| 34 | 66 | 24.1 | 20.4 | 39.7 | 40.3 | 41.4 | 29.1 | 38 | 59.5 | 114.0 | 26.4 |
| 35 | 75 | 111 | 51.3 | 201.6 | 116.3 | 89.5 | 51 | 84 | 64.1 | 74.7 | 57.6 |
| 36 | 95 | 28.6 | 62.5 | 76.2 | 93.6 | 95.6 | 75 | 59 | 71.6 | 56.3 | 69.2 |
| 37 | 56 | 4.1 | 16.5 | 50.8 | 54.9 | 51.9 | 29 | 53 | 40.9 | 35.1 | 39.2 |
| 38 | 53 | 55.7 | 22.5 | 54.8 | 55.9 | 54 | 38.3 | 59 | 40.1 | 37.9 | 41 |
| 39 | 59 | 14.5 | 25.3 | 62.2 | 71.8 | 61.7 | 20.9 | 78 | 55.4 | 38.3 | 49 |
| 40 | 46 | 76 | 43.5 | 58 | 74 | 74.4 | 26 | 48 | 59.3 | 33.5 | 43.8 |
| 41 | 30.5 | 23.8 | 24.2 | 86.4 | 52.7 | 49.2 | 29.6 | 42 | 42.2 | 30.3 | 25.6 |
| 42 | 218 | 116 | 63.6 | 148.3 | 198.6 | 204.2 | 366 | 213 | 173 | 205.0 | 29.8 |
| 43 | 91 | 15.5 | 36.9 | 175.2 | 243.9 | 224.3 | 241 | 614 | 185 | 82.7 | 65.6 |
|  |  |  |  |  |  |  |  |  |  |  |  |
| 44 | 119 | 15.1 | 78.4 | 150.8 | 136.5 | 129.3 | 301 | 94 | 99.1 | 112.0 | 83.2 |
