# Supplementary material for: Inter-laboratory comparison of eleven quantitative or digital PCR assays for detection of proviral bovine leukemia virus in blood samples
Source: BMC Vet Res. 2024 Aug 26;20:381. doi: 10.1186/s12917-024-04228-z (PMC11346035; doi:10.1186/s12917-024-04228-z)
Supplement: Supplementary file 4 — Additional file 4. Post hoc - Dunn-Bonferroni-Tests. The Dunn-Bonferroni test revealed that the pairwise group comparisons of qPCR2 - qPCR4, qPCR3 - ddPCR6, qPCR4 - qPCR5, qPCR4 - ddPCR6, qPCR4 - qPCR9, qPCR4 - qPCR10, qPCR5 - qPCR11, ddPCR6 - qPCR11 and qPCR9 - qPCR11 have an adjusted p-value less than 0,05 [file 12917_2024_4228_MOESM4_ESM.pdf]

Additional file 2. Post hoc - Dunn-Bonferroni-Tests. The Dunn-Bonferroni test revealed that the pairwise group comparisons of qPCR2 - qPCR4, qPCR3 - ddPCR6, qPCR4 - qPCR5, qPCR4 - ddPCR6, qPCR4 - qPCR9, qPCR4 - qPCR10, qPCR5 - qPCR11, ddPCR6 - qPCR11 and qPCR9 - qPCR11 have an adjusted p-value less than 0,05.

	Test Statistic	Std. Error	Std. Test Statistic	p	Adj. p
qPCR1 - qPCR2	25.84	28.76	0.9	.369	1
qPCR1 - qPCR3	-34.22	28.76	-1.19	.234	1
qPCR1 - qPCR4	-78.77	28.76	-2.74	.006	.339
qPCR1 - qPCR5	27.09	28.76	0.94	.346	1
qPCR1 - ddPCR6	66.01	28.76	2.3	.022	1
qPCR1 - qPCR7	-3.1	28.76	-0.11	.914	1
qPCR1 - qPCR8	14.12	28.76	0.49	.623	1
qPCR1 - qPCR9	58.26	28.76	2.03	.043	1
qPCR1 - qPCR10	20.22	28.76	0.7	.482	1
qPCR1 - qPCR11	-68.76	28.76	-2.39	.017	.926
qPCR2 - qPCR3	-60.06	28.76	-2.09	.037	1
qPCR2 - qPCR4	-104.61	28.76	-3.64	<.001	.015
qPCR2 - qPCR5	1.24	28.76	0.04	.966	1
qPCR2 - ddPCR6	40.17	28.76	1.4	.163	1
qPCR2 - qPCR7	-28.94	28.76	-1.01	.314	1
qPCR2 - qPCR8	-11.72	28.76	-0.41	.684	1
qPCR2 - qPCR9	32.41	28.76	1.13	.26	1
qPCR2 - qPCR10	-5.62	28.76	-0.2	.845	1
qPCR2 - qPCR11	-94.6	28.76	-3.29	.001	.055
qPCR3 - qPCR4	-44.55	28.76	-1.55	.121	1
qPCR3 - qPCR5	61.3	28.76	2.13	.033	1
qPCR3 - ddPCR6	100.23	28.76	3.48	<.001	.027
qPCR3 - qPCR7	31.12	28.76	1.08	.279	1
qPCR3 - qPCR8	48.34	28.76	1.68	.093	1
qPCR3 - qPCR9	92.48	28.76	3.22	.001	.072
qPCR3 - qPCR10	54.44	28.76	1.89	.058	1
qPCR3 - qPCR11	-34.54	28.76	-1.2	.23	1
qPCR4 - qPCR5	105.85	28.76	3.68	<.001	.013
qPCR4 - ddPCR6	144.78	28.76	5.03	<.001	<.001
qPCR4 - qPCR7	75.67	28.76	2.63	.009	.468
qPCR4 - qPCR8	92.89	28.76	3.23	.001	.068
qPCR4 - qPCR9	137.02	28.76	4.76	<.001	<.001

qPCR4 - qPCR10	98.99	28.76	3.44	.001	.032
qPCR4 - qPCR11	10.01	28.76	0.35	.728	1
qPCR5 - ddPCR6	38.93	28.76	1.35	.176	1
qPCR5 - qPCR7	-30.18	28.76	-1.05	.294	1
qPCR5 - qPCR8	-12.96	28.76	-0.45	.652	1
qPCR5 - qPCR9	31.17	28.76	1.08	.278	1
qPCR5 - qPCR10	-6.87	28.76	-0.24	.811	1
qPCR5 - qPCR11	-95.84	28.76	-3.33	.001	.047
ddPCR6 - qPCR7	-69.11	28.76	-2.4	.016	.895
ddPCR6 - qPCR8	-51.89	28.76	-1.8	.071	1
ddPCR6 - qPCR9	-7.76	28.76	-0.27	.787	1
ddPCR6 - qPCR10	-45.79	28.76	-1.59	.111	1
ddPCR6 - qPCR11	-134.77	28.76	-4.69	<.001	<.001
qPCR7 - qPCR8	17.22	28.76	0.6	.549	1
qPCR7 - qPCR9	61.35	28.76	2.13	.033	1
qPCR7 - qPCR10	23.32	28.76	0.81	.418	1
qPCR7 - qPCR11	-65.66	28.76	-2.28	.022	1
qPCR8 - qPCR9	44.13	28.76	1.53	.125	1
qPCR8 - qPCR10	6.1	28.76	0.21	.832	1
qPCR8 - qPCR11	-82.88	28.76	-2.88	.004	.218
qPCR9 - qPCR10	-38.04	28.76	-1.32	.186	1
qPCR9 - qPCR11	-127.01	28.76	-4.42	<.001	.001
qPCR10 - qPCR11	-88.98	28.76	-3.09	.002	.109

Adj. p: Values adjusted with Bonferroni correction.
