# Supplementary material for: Inter-laboratory comparison of eleven quantitative or digital PCR assays for detection of proviral bovine leukemia virus in blood samples
Source: BMC Vet Res. 2024 Aug 26;20:381. doi: 10.1186/s12917-024-04228-z (PMC11346035; doi:10.1186/s12917-024-04228-z)
Supplement: Supplementary file 5 — Additional file 5. Kendall's Tau correlation coefficient values measured between each pair of assays. The numbers 1 to 11 in the first column and last row of the table indicate the names of the assays qPCR1-qPCR5, ddPCR6, qPCR7-qPCR11 respectively [file 12917_2024_4228_MOESM5_ESM.docx]

**Additional file 5.** Kendall's Tau correlation coefficient values measured between each pair of assays. The numbers 1 to 11 in the first column and last row of the table indicate the names of the assays qPCR1-qPCR5, ddPCR6, qPCR7-qPCR11 respectively.

| 11 | 0.818792 | 0.655586 | 0.588692 | 0.814648 | 0.793399 | 0.741593 | 0.787287 | 0.789732 | 0.772617 | 0.80903 | 1.0 |
| --- | --- | --- | --- | --- | --- | --- | --- | --- | --- | --- | --- |
| 10 | 0.829268 | 0.652704 | 0.582179 | 0.824035 | 0.783141 | 0.708313 | 0.707393 | 0.709836 | 0.785585 | 1.0 | 0.80903 |
| 9 | 0.824681 | 0.662603 | 0.633803 | 0.865153 | 0.822521 | 0.680519 | 0.734394 | 0.805386 | 1.0 | 0.785585 | 0.772617 |
| 8 | 0.814907 | 0.677521 | 0.627637 | 0.806981 | 0.793146 | 0.646367 | 0.78825 | 1.0 | 0.805386 | 0.709836 | 0.789732 |
| 7 | 0.736715 | 0.66136 | 0.625171 | 0.790891 | 0.705018 | 0.717202 | 1.0 | 0.78825 | 0.734394 | 0.707393 | 0.787287 |
| 6 | 0.690637 | 0.558851 | 0.647344 | 0.767445 | 0.648897 | 1.0 | 0.717202 | 0.646367 | 0.680519 | 0.708313 | 0.741593 |
| 5 | 0.873551 | 0.696169 | 0.567217 | 0.800792 | 1.0 | 0.648897 | 0.705018 | 0.793146 | 0.822521 | 0.783141 | 0.793399 |
| 4 | 0.799326 | 0.668768 | 0.642149 | 1.0 | 0.800792 | 0.767445 | 0.790891 | 0.806981 | 0.865153 | 0.824035 | 0.814648 |
| 3 | 0.606795 | 0.495946 | 1.0 | 0.642149 | 0.567217 | 0.647344 | 0.625171 | 0.627637 | 0.633803 | 0.582179 | 0.588692 |
| 2 | 0.657667 | 1.0 | 0.495946 | 0.668768 | 0.696169 | 0.558851 | 0.66136 | 0.677521 | 0.662603 | 0.652704 | 0.655586 |
| 1 | 1.0 | 0.657667 | 0.606795 | 0.799326 | 0.873551 | 0.690637 | 0.736715 | 0.814907 | 0.824681 | 0.829268 | 0.818792 |
|  | 1 | 2 | 3 | 4 | 5 | 6 | 7 | 8 | 9 | 10 | 11 |
