# Supplementary material for: Inter-laboratory comparison of eleven quantitative or digital PCR assays for detection of proviral bovine leukemia virus in blood samples
Source: BMC Vet Res. 2024 Aug 26;20:381. doi: 10.1186/s12917-024-04228-z (PMC11346035; doi:10.1186/s12917-024-04228-z)
Supplement: Supplementary file 7 — Additional file 7. Multiple sequence alignment of reverse transcriptase, integrase, envelope and LTR sequences in the context of the specific primers used by different qPCR assays. (A) Multiple sequence alignment of reverse transcriptase (pol gene) sequences in the context of qPCR7, qPCR8, qPCR9, qPCR10 and qPCR11 assay primers. (B) Multiple sequence alignment of integrase (pol gene) sequences in the context of qPCR4 assay primers. (C) Multiple sequence alignment of env gene sequences in the context of ddPCR6. (D) Sequence alignment of LTR region sequences in the context of qPCR3 method primers [file 12917_2024_4228_MOESM7_ESM.pdf]

A

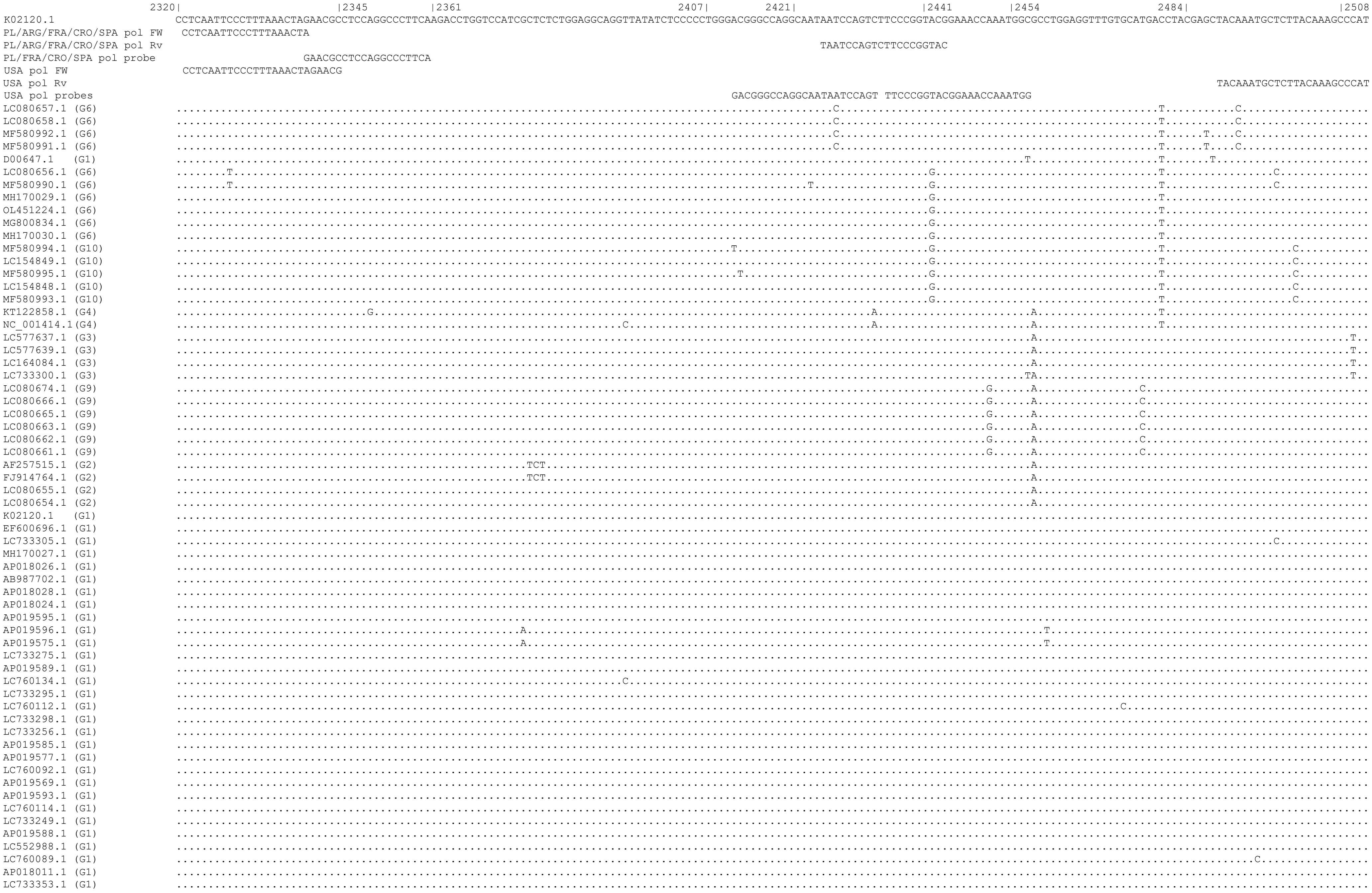
2320| |2345 |2361 2407| 2421| |2441 |2454 2484| |2508
K02120.1 CCTCAATTCCCTTTAAACTAGAACGCCTCCAGGCCCTTCAAGACCTGGTCCATCGCTCTCTGGAGGCAGGTTATATCTCCCCCTGGGACGGGCCAGGCAATAATCCAGTCTTCCCGGTACGGAAACCAAATGGCGCCTGGAGGTTTGTGCATGACCTACGAGCTACAAATGCTCTTACAAAGCCCAT
PL/ARG/FRA/CRO/SPA pol FW CCTCAATTCCCTTTAAACTA
PL/ARG/FRA/CRO/SPA pol Rv TAATCCAGTCTTCCCGGTAC
PL/FRA/CRO/SPA pol probe GAACGCCTCCAGGCCCTTCA
USA pol FW CCTCAATTCCCTTTAAACTAGAACG
USA pol Rv TACAAATGCTCTTACAAAGCCCAT
USA pol probes GACGGGCCAGGCAATAATCCAGT TTCCCGGTACGGAAACCAAATGG
LC080657.1 (G6)
LC080658.1 (G6)
MF580992.1 (G6)
MF580991.1 (G6)
D00647.1 (G1)
LC080656.1 (G6)
MF580990.1 (G6)
MH170029.1 (G6)
OL451224.1 (G6)
MG800834.1 (G6)
MH170030.1 (G6)
MF580994.1 (G10)
LC154849.1 (G10)
MF580995.1 (G10)
LC154848.1 (G10)
MF580993.1 (G10)
KT122858.1 (G4)
NC_001414.1 (G4)
LC577637.1 (G3)
LC577639.1 (G3)
LC164084.1 (G3)
LC733300.1 (G3)
LC080674.1 (G9)
LC080666.1 (G9)
LC080665.1 (G9)
LC080663.1 (G9)
LC080662.1 (G9)
LC080661.1 (G9)
AF257515.1 (G2)
FJ914764.1 (G2)
LC080655.1 (G2)
LC080654.1 (G2)
K02120.1 (G1)
EF600696.1 (G1)
LC733305.1 (G1)
MH170027.1 (G1)
AP018026.1 (G1)
AB987702.1 (G1)
AP018028.1 (G1)
AP018024.1 (G1)
AP019595.1 (G1)
AP019596.1 (G1)
AP019575.1 (G1)
LC733275.1 (G1)
AP019589.1 (G1)
LC760134.1 (G1)
LC733295.1 (G1)
LC760112.1 (G1)
LC733298.1 (G1)
LC733256.1 (G1)
AP019585.1 (G1)
AP019577.1 (G1)
LC760092.1 (G1)
AP019569.1 (G1)
AP019593.1 (G1)
LC760114.1 (G1)
LC733249.1 (G1)
AP019588.1 (G1)
LC552988.1 (G1)
LC760089.1 (G1)
AP018011.1 (G1)
LC733353.1 (G1)

```
LC733321.1 (G1)            ............................................................................................................................................................................................
LC080652.1 (G1)            ............................................................................................................................................................................................
LC080651.1 (G1)            ............................................................................................................................................................................................
HE967301.1 (G1)            ............................................................................................................................................................................................
HE967303.1 (G1)            ............................................................................................................................................................................................
HE967302.1 (G1)            ............................................................................................................................................................................................
LC733309.1(G1)             ....................................................................................................C.......................................................................................
LC733294.1(G1)             ............................................................................................................................................................................................
LC733277.1(G1)             ............................................................................................................................................................................................
LC552971.1(G1)             .....................................................................................................................................T......................................................
LC552993.1(G1)             ............................................................................................................................................................................................
LC760131.1(G1)             .....................................................................................................................................T.....................T................................
LC760135.1(G1)             ............................................................................................................................................................................C...............
LC733319.1(G1)             ............................................................................................................................................................................................
AP019582.1(G1)             ............................................................................................................................................................................................
AP019572.1(G1)             ............................................................................................................................................................................................
AB934282.1(G1)             ............................................................................................................................................................................................
LC733265.1(G1)             ............................................................................................................................................................................................
LC552987.1(G1)             ............................................................................................................................................................................................
LC552986.1(G1)             .........................................................................C..................................................................................................................
LC552976.1(G1)             ............................................................................................................................................................................................
LC552968.1(G1)             ............................................................................................................................................................................................
LC733335.1(G1)             ............................................................................................................................................................................................
LC733349.1(G1)             ............................................................................................................................................................................................
AP018023.1(G1)             ............................................................................................................................................................................................
LC733334.1(G1)             ............................................................................................................................................................................................
LC760127.1(G1)             ............................................................................................................................................................................................
LC760125.1(G1)             ............................................................................................................................................................................................
LC733343.1(G1)             ............................................................................................................................................................................................
LC733333.1(G1)             ............................................................................................................................................................................................
LC681771.1(G1)             ............................................................................................................................................................................................
LC005615.1(G1)             ............................................................................................................................................................................................
LC760106.1(G1)             ............................................................................................................................................................................................
MH170028.1(G1)             ............................................................................................................................................................................................
AP018009.1(G1)             ............................................................................................................................................................................................
LC760098.1(G1)             ............................................................................................................................................................................................
AP018019.1(G1)             ................................................A.................................................................................T.....................................................
KP113663.1(G1)             .......................................................................................................................................................................................
```

B

```
K02120.1                 CACCATTCACCCCCACTTGCTGTCATTTCAGAGGGCGGAGAAACACCCAAGGGCTCTGA
ARG pol FW               CACCATTCACCCCCACTTG
ARG pol Rv                                                      GAAACACCCAAGGGCTCTGA
LC080657.1 (G6)          ...............................................T...........
LC080658.1 (G6)          ...............................................T...........
MF580992.1 (G6)          ...............................................T...........
MF580991.1 (G6)          ...............................................T...........
D00647.1   (G1)          ...........................................................
LC080656.1 (G6)          ...........................................................
MF580990.1 (G6)          ...............................................A...........
MH170029.1 (G6)          ...........................................................
OL451224.1 (G6)          ...........................................................
MG800834.1 (G6)          ...........................................................
MH170030.1 (G6)          ...........................................................
MF580994.1 (G10)         ...........................................................
LC154849.1 (G10)         ...........................................................
MF580995.1 (G10)         ...........................................................
LC154848.1 (G10)         ...........................................................
MF580993.1 (G10)         ...........................................................
KT122858.1 (G4)          ...........................................................
NC_001414.1(G4)          ...........................................................
LC577637.1 (G3)          ...........T...............................................
LC577639.1 (G3)          ...........T...............................................
LC164084.1 (G3)          ...........................................................
LC733300.1 (G3)          .........................................C.................
LC080674.1 (G9)          ...........................................................
LC080666.1 (G9)          ...........................................................
LC080665.1 (G9)          ...........................................................
LC080663.1 (G9)          ...........................................................
LC080662.1 (G9)          ...........................................................
LC080661.1 (G9)          ...........................................................
AF257515.1 (G2)          ...........................................................
FJ914764.1 (G2)          ...........................................................
LC080655.1 (G2)          ...........................................................
LC080654.1 (G2)          ...........................................................
K02120.1   (G1)          ...........................................................
EF600696.1 (G1)          ...........................................................
LC733305.1 (G1)          ...........................................................
MH170027.1 (G1)          ...........................................................
AP018026.1 (G1)          ...........................................................
AB987702.1 (G1)          ...........................................................
AP018028.1 (G1)          ...........................................................
AP018024.1 (G1)          ...........................................................
AP019595.1 (G1)          ...........................................................
AP019596.1 (G1)          ...........................................................
AP019575.1 (G1)          ...........................................................
LC733275.1 (G1)          ...........................................................
AP019589.1 (G1)          ...........................................................
LC760134.1 (G1)          ...........................................................
LC733295.1 (G1)          ...........................................................
LC760112.1 (G1)          ...........................................................
LC733298.1 (G1)          ...........................................................
LC733256.1 (G1)          ...........................................................
AP019585.1 (G1)          ...........................................................
AP019577.1 (G1)          ...........................................................
LC760092.1 (G1)          ...........................................................
AP019569.1 (G1)          ...........................................................
AP019593.1 (G1)          ...........................................................
LC760114.1 (G1)          ...........................................................
LC733249.1 (G1)          ...........................................................
AP019588.1 (G1)          ...........................................................
LC552988.1 (G1)          ...........................................................
LC760089.1 (G1)          ...........................................................
AP018011.1 (G1)          ...........................................................
LC733353.1 (G1)          ...........................................................
LC733321.1 (G1)          ...........................................................
LC080652.1 (G1)          ...........................................................
LC080651.1 (G1)          ...........................................................
HE967301.1 (G1)          ...........................................................
HE967303.1 (G1)          ...........................................................
HE967302.1 (G1)          ...........................................................
LC733309.1 (G1)          ...........................................................
LC733294.1 (G1)          ...........................................................
LC733277.1 (G1)          ...........................................................
LC552971.1 (G1)          ...........................................................
LC552993.1 (G1)          ...........................................................
LC760131.1 (G1)          ...........................................................
LC760135.1 (G1)          ...........................................................
LC733319.1 (G1)          ...........................................................
AP019582.1 (G1)          ...........................................................
AP019572.1 (G1)          ...........................................................
AB934282.1 (G1)          ...........................................................
LC733265.1 (G1)          ...........................................................
LC552987.1 (G1)          ...........................................................
LC552986.1 (G1)          ...........................................................
LC552976.1 (G1)          ...........................................................
LC552968.1 (G1)          ...........................................................
LC733335.1 (G1)          ...........................................................
LC733349.1 (G1)          ...........................................................
AP018023.1 (G1)          ...........................................................
LC733334.1 (G1)          ...........................................................
LC760127.1 (G1)          ...........................................................
LC760125.1 (G1)          ...........................................................
LC733343.1 (G1)          ...........................................................
LC733333.1 (G1)          ...........................................................
LC681771.1 (G1)          ...........................................................
LC005615.1 (G1)          ...........................................................
LC760106.1 (G1)          ...........................................................
MH170028.1 (G1)          ...........................................................
AP018009.1 (G1)          ...........................................................
LC760098.1 (G1)          ...........................................................
AP018019.1 (G1)          ...........................................................
KP113663.1 (G1)          ...........................................................
```

C

```
             5384|                      |5406                                                             5477|                    |5497                  5525|                     |5546
K02120.1          CAGTGACTGGGTTCCCTCTGTCAGATCATGGGCCCTGCTTTTAAATCAAACAGCACGGGCCTTCCCAGACTGTGCTATATGTTGGGAACCTTCCCCTCCCTGGGCTCCCGAAATATTAGTATATAACAAAACCATCTCCAGCTCTGGACCCGGCCTCGCCCT
ddPCR env FW      CAGTGACTGGGTTCCCTCTGTC
ddPCR env Rv                                                                                                                                                                     CTCTGGACCCGGCCTCGCCCT
ddPCR env probe                                                                                                CCCTCCCTGGGCTCCCGAAA
LC080654.1 (G2)   ........................................C.......G..G..............................................................................................................
FJ914764.1 (G2)   ................................................G..G..............................................................................................................
FJ808593.1 (G2)   ................................................G..G..............................................................................................................
FJ808577.1 (G2)   ................................................G..G...............................................................G..............................................
FJ808579.1 (G2)   ................................................G..G..............................................................................................................
AF257515.1 (G2)   ................................................G..G..............................................................................................................
LC760094.1 (G1)   ...................................................C..............................................................................................................
LC760092.1 (G1)   .............................................C.....C..............................................................................................................
AP019577.1 (G1)   .............................................C.....C..............................................................................................................
AP019582.1 (G1)   ...........................................................................................................................................G......................
AP018022.1 (G1)   ................................................................................................................................G.................................
AP019598.1 (G1)   T.................................................................................................................................................................
LC007986.1 (G1)   ..................................................................................................................................................................
LC552974.1 (G1)   ..................................................................................................................................................................
LC552976.1 (G1)   ..................................................................................................................................................................
MW926783.1 (G1)   ..................................................................................................................................................................
LC775095.1 (G1)   ..................................................................................................................................................................
K02120.1   (G1)   ..................................................................................................................................................................
LC361259.1 (G3)   ...................................................G.......................C...........G..........................................................................
LC164084.1 (G3)   ...................................................G.......................C...........G..........................................................................
LC007993.1 (G3)   ...................................................G.......................C...........G..........................................................................
LC733284.1 (G3)   ...................................................G.......................C...........G..........................................................................
LC733283.1 (G3)   ..A................................................G.......................C...........G..........................................................................
EF065650.1 (G3)   ...................................................G.......................C...........G..........................................................................
LC733300.1 (G3)   ...................................................G.......................C...........G..........................................................................
LC080666.1 (G9)   ...................................................G....................C.........................................................................................
LC080659.1 (G9)   ...................................................G....................C.........................................................................................
LC080674.1 (G9)   ...................................................G....................C.........................................................................................
LC080673.1 (G9)   ...................................................G....................C.........................................................................................
LC080675.1 (G9)   ...................................................G....................C.........................................................................................
LC080672.1 (G9)   ...................................................G....................C.........................................................................................
LC080663.1 (G9)   ...................................................G....................C.........................................................................................
JQ675760.1 (G8)   .................................T.................G........................................................T.....................................................
HM563764.3 (G8)   .................................T.................G........................................................T................................T..C.................
JQ675759.1 (G8)   .................................T.................G.........................................T..............T...................................C.................
MK840880.1 (G6)   ..A................................................G.....A........................................................................................................
MH341520.1 (G6)   ......T...........................T................G..............................................................................................................
MH341523.1 (G6)   ......T............................................G..............................................................................................................
MH341519.1 (G6)   ......T...........................T................G..............................................................................................................
MW926787.1 (G6)   .............................................C.....G..............................................................................................................
LC512449.1 (G6)   ...................................................G..............................................................................................................
MH170029.1 (G6)   ...................................................G..............................................................................................................
MF574056.1 (G6)   ......T............................................G...................................C..........................................................................
MF574054.1 (G6)   ...................................................G...................................G..........................................................................
LC080656.1 (G6)   ......T............................................G..............................................................................................................
MF574053.1 (G6)   ......T............................................G..............................................................................................................
MF580991.1 (G6)   ......T............................................G..............................................................................................................
KU233543.1 (G10)  ...................................................G...A.................................................................................................T........
LC606682.1 (G10)  T..................................................G...A.................................................................................................T........
LC606674.1 (G10)  T..................................................G...A.................................................................................................T........
LC154066.1 (G10)  T...................................A..............G...A.......................................................C.........................................T........
LC154849.1 (G10)  T..................................................G..GA..........C......................................................................................T........
LC154065.1 (G10)  T..................................................G..GA..........C......................................................................................T........
MF580994.1 (G10)  T..................................................G..GA.................................................................................................T........
LC512452.1 (G10)  T..................................................G...A.................................................................................................T........
EF065643.1 (G5)   .......................A................C..........G...................................G......................................T..........................T........
EF065635.1 (G5)   ........................................C..........G..........................................................................T..........................T........
EF065655.1 (G5)   ........................G...............C..........G...........................................................G..............T..........................T........
EF065639.1 (G5)   ........................................C..........G..........................................................................T..........................T........
EF065654.1 (G5)   ........................................C....C.....G..........................................................................T..........................T........
```

```
OK945991.1 (G4)   ...........................................................................................................................................G.............T........
HM563783.3 (G4)   ...................................................G.....................................................................................................T........
OK945987.1 (G4)   ...................................................G...............................................................C.....................................T........
KT122858.1 (G4)   ........................G..........................G........................................................................................A............T........
JQ686092.1 (G4)   ...................................................G.....................................................................................................T........
AF033818.1 (G4)   ...................................................G.......................................................................................G.............T........
K02251.1   (G4)   ...................................................G.......................................................................................G.............T........
JQ686089.1 (G4)   ...................................................G..............T........................................................................G.............T........
JQ686090.1 (G4)   ...................................................G..............T........................................................................G.............T........
JN695878.1 (G4)   ...................................................G.....................................................................................................T........
JQ353644.1 (G4)   ..................C................................G.....................................................................................................T........
MN966691.1 (G4)   ...................................................G.....................................................................................................T........
OK945981.1 (G4)   ...................................................G.....................................................................................................T........
OL660381.1 (G4)   ...................................................G.....................................................................................................T........
OP850790.1 (G4)   ...................................................G....................C................................................................................T........
OL660339.1 (G4)   ...................................................G.....................................................................................................T........
OL660295.1 (G4)   ...................................................G.....................................................................................................T........
OL660291.1 (G4)   ...................................................G.....................................................................................................T........
OL660308.1 (G4)   ...................................................G.....................................................................................................T........
OL660261.1 (G4)   ...................................................G...............................................T..........................T..........................T........
OP850719.1 (G4)   ...................................................G.....................................................................................................T........
OP850710.1 (G4)   ...................................................G.....................................................................................................T........
OL660266.1 (G4)   ...................................................G.....................................................................................................T........
ON799094.1 (G4)   ...................................................G.....................................................................................................T........
OK945977.1 (G4)   ...................................................G...........................................................G.........................................T........
OP850727.1 (G4)   ...................................................G...........................................................R...........Y.............................T........
S83530.1   (G7)   ...................................................G.........................................T.................G.........................................T........
MN765155.1 (G7)   ...................................................G.......................................................................C.............................T........
JQ353653.1 (G7)   ...................................................G...........................................................G...........C..........C..................T........
HM563758.3 (G7)   ...................................................G...........................................................G...........C.............................T........
JF720353.2 (G7)   ...................................................G...........................................................G...........C......................................
KF801461.2 (G7)   ...................................................G...........................................................G...........C.............................T........
OK945983.1 (G7)   T..................................................G...........................................................G...........C.............................T........
OL660334.1 (G7)   ...................................................G...........................................................G...........C.............................T........
OL660400.1 (G7)   ...................................................G...........................................................G..G........C.............................T........
OL660398.1 (G7)   ...................................................G...........................................................G..G........C.............................T........
OL660397.1 (G7)   ...................................................G...........................................................G...........C.............................T........
OL660394.1 (G7)   ...................................................G...........................................................G...........C.............................T........
JN695882.1 (G7)   ...................................................G...........................................................G...........C.............................T........
JQ686116.1 (G7)   ...................................................G...........................................................G...........C.............................T........
```

D

```
Position          37|                 |54                                             108|                                            |150
K02120.1             GCCCCGTAAACCAGACAGAGACGTCAGCTGCCAGAAAAGCTGGTGACGGCAGCTGGTGGCTAGAATCCCCGTACCTCCCCAACTTCCCCTTTCCCGAAAAATCCACACCCTGAG
BRA LTR FW           GCCCCGTAAACCAGACAG
BRA LTR Rv                                                                                                         CGAAAAATCCACACCCTGAG
BRA LTR probe                                                                               TACCTCCCCAACTTCCCCTTTCC
MH170030.1 (G6)      ......CG...............................................................C..........................................
MH170029.1 (G6)      ......CG...............................................................C..........................................
MG800834.1 (G6)      ......CG...............................................................C.........................G................
LC080658.1 (G6)      ......C................................................................C.........................G................
LC080657.1 (G6)      ......C................................................................C.........................G................
MF580992.1 (G6)      ......C................................................................C.........................G.........G......
AF257515.1 (G2)      ....T..............A..............................................................................................
FJ914764.1 (G2)      ....T..............A..............................................................................................
LC080670.1 (G9)      ....T.............................................................................................................
LC080672.1 (G9)      ....T.............................................................................................................
LC080667.1 (G9)      ....T.............................................................................................................
LC080674.1 (G9)      ....T.............................................................................................................
LC577640.1 (G3)      ....T.............................................................................................................
LC733300.1 (G3)      ....T.............................................................................................................
DQ288183.1 (G3)      ....T...........................................................................G.................................
LC577637.1 (G3)      ....T...........................................................................G.................................
MF580994.1 (G10)     .......G...............................................................C.........................G................
MF580993.1 (G10)     .......G...............................................................C.........................G...C............
LC154848.1 (G10)     .......G...............................................................C.........................G................
LC154849.1 (G10)     .......G...............................................................C.........................G................
MF580995.1 (G10)     .......G...............................................................C.........................G................
AH002557.2 (G4)      ...................................G......C................TC.....................................................
MG407619.1 (G4)      ..........................................A.......................................................................
MH423673.1 (G4)      ..........................................A.......................................................................
MW256517.1 (G4)      ..........................................A.......................................................................
MW256515.1 (G4)      ..........................................A.......................................................................
MW256514.1 (G4)      ..........................................A.......................................................................
MG407618.1 (G7)      ..........................................C.......................................................................
MW256551.1 (G7)      ..........................................C.......................................................................
MW256546.1 (G7)      ..........................................C.......................................................................
MT740115.1 (G4)      ....T.C.......................................................................................................C...
MH407739.1 (G7)      ....................................................................................................-AT...........
MT740121.1 (G4)      ..................................G.G.........................................................................C...
MT740146.1 (G8)      .....A........................................................................................................C...
MH423639.1 (G8)      .....A........................................................................................................C...
MH423653.1 (G8)      .....A........................................................................................................C...
MT740142.1 (G8)      .....A........................................................................................................C...
MT740143.1 (G8)      .....A........................................................................................................C...
MH423652.1 (G8)      .....A........................................................................................................C...
MT740137.1 (G8)      .....A........................................................................................................C...
MH423636.1 (G4)      ..............................................................................................................C...
MT740113.1 (G4)      ..............................................................................................................C...
MH423634.1 (G4)      ..............................................................................................................C...
MT740112.1 (G4)      ..............................................................................................................C...
LC733280.1 (G1)      .............................A..................................................C.................................
LC733252.1 (G1)      .............................A..................................................C.................................
LC733292.1 (G1)      ................................................................................C.................................
```

LC733283.1 (G3)	....T...
DQ288222.1 (G5)	....G................
MW256539.1 (G4)	....C...
MH423647.1 (G4)	....C...
MW256532.1 (G4)	.....A..
MH423678.1 (G4)	...................................G..
MW256540.1 (G4)	.................................A..
DQ288177.1 (G1)	....A..
MH423638.1 (G7)	....C..
LC733272.1 (G1)	................................T...
AP019591.1 (G1)	....C..
LC682198.1 (G1)	....
DQ287264.1 (G1)	....A...A..
LC733257.1 (G1)	....A..
OL804086.1 (G1)	....A.......................................
MW256526.1 (G1)	....T...................
HE967303.1 (G1)	....A..
AP018009.1 (G1)	.....................................G..
AB934282.1 (G1)	.....................................G..
LC760137.1 (G1)	................................C...
AP019570.1 (G1)	....T......
LC552977.1 (G1)	A...
MW256552.1 (G1)	....
MT740155.1 (G1)	....
MT740153.1 (G1)	....
OL804077.1 (G1)	....
LC733363.1 (G1)	....
LC733246.1 (G1)	....
LC552979.1 (G1)	....
LC552973.1 (G1)	....
OL804098.1 (G1)	....
AP019598.1 (G1)	....
AP019577.1 (G1)	....
OL804082.1 (G1)	....
OL804080.1 (G1)	....
LC733262.1 (G1)	....
LC733318.1 (G1)	....
LC733328.1 (G1)	....
LC733340.1 (G1)	....
LC733315.1 (G1)	....
LC733278.1 (G1)	....
OL804094.1 (G1)	....
AP018023.1 (G1)	....
AP018021.1 (G1)	....
LC728442.1 (G1)	....
AH002328.2 (G1)	....
K02120.1 (G1)	....
AH001143.2 (G1)	....
MW256531.1 (G4)	....
MH423669.1 (G4)	....
MH423642.1 (G4)	....
MT740101.1 (G4)	....
MT740103.1 (G4)	....
